# Thymoquinone Potentiates Docetaxel-Induced Antitumor Activity with the Involvement of ROS and PI3K/AKT Pathway Modulation in Triple-Negative Breast Cancer Cells

**DOI:** 10.3390/ph19081154

**Published:** 2026-07-24

**Authors:** Aylin Orhaner, Mehmet Cudi Tuncer, İlhan Özdemir

**Affiliations:** 1 Department of Gynecology and Obstetrics, Medicana Bursa Hospital, 16150 Bursa, Turkey; 2 Department of Anatomy, Faculty of Medicine, Dicle University, 21280 Diyarbakır, Turkey; drcudi@hotmail.com; 3 Department of Histology and Embryology, Faculty of Medicine, Kahramanmaraş Sütçü İmam University, 46000 Kahramanmaraş, Turkey; ilhanozdemir32@hotmail.com

**Keywords:** thymoquinone, docetaxel, triple-negative breast cancer, MDA-MB-231, apoptosis, combination index

## Abstract

**Background**: Drug resistance and treatment-associated toxicity remain major limitations of conventional chemotherapy for triple-negative breast cancer (TNBC). Thymoquinone (TQ), a bioactive phytochemical derived from *Nigella sativa*, has demonstrated anticancer properties and may enhance the therapeutic efficacy of docetaxel (DTX) through complementary molecular mechanisms. **Objective**: To investigate whether TQ potentiates the antitumor activity of DTX in MDA-MB-231 TNBC cells by affecting apoptosis, oxidative stress, wound closure, and PI3K/AKT pathway-related gene expression. **Methods**: MDA-MB-231 TNBC cells and HaCaT keratinocytes were treated with TQ, DTX, or their combination. Cell viability was determined using the MTT assay, and drug interactions were evaluated by the Chou–Talalay combination index (CI) method. Apoptosis, intracellular reactive oxygen species (ROS) production, ROS rescue experiments using N-acetyl-L-cysteine (NAC), caspase-9 expression, wound closure, and gene-expression changes were assessed using Annexin V/PI flow cytometry, DCFH-DA-based flow cytometric and fluorescence analyses, immunocytochemistry, wound-healing assay, and quantitative real-time PCR (qRT-PCR), respectively. Bioinformatic analyses were performed to identify signaling pathways associated with the observed molecular alterations. **Results**: The TQ + DTX combination demonstrated synergistic cytotoxicity and significantly increased apoptotic cell death compared with either monotherapy. Combination treatment markedly enhanced intracellular ROS accumulation, whereas NAC pretreatment significantly attenuated ROS generation and partially reversed the cytotoxic and pro-apoptotic effects, suggesting the involvement of ROS in the observed antitumor effects. Caspase-9 immunoreactivity was markedly increased following combination treatment, suggesting the involvement of the intrinsic apoptotic pathway. Furthermore, the combination significantly suppressed wound closure and downregulated *BCL2*, *PIK3CA*, and *AKT1* while upregulating *BAX*, *CASP9*, and *PTEN*. Bioinformatic analyses identified apoptosis, p53, PI3K/AKT, mTOR, and MAPK signaling as the principal pathways potentially associated with the observed gene expression changes. **Conclusions**: TQ potentiates the antitumor activity of DTX, with the involvement of oxidative stress, apoptotic signaling, suppression of wound closure, and regulation of PI3K/AKT pathway-related gene expression in TNBC cells. These findings provide evidence supporting further preclinical investigation of the TQ + DTX combination as a promising therapeutic strategy for triple-negative breast cancer.

## 1. Introduction

Breast cancer remains the most commonly diagnosed malignancy among women worldwide and continues to impose a substantial global health burden, with more than 2.3 million new cases reported annually [1,2]. Among its molecular subtypes, TNBC is recognized as one of the most aggressive forms due to the absence of estrogen receptor (ER), progesterone receptor (PR), and human epidermal growth factor receptor 2 (HER2) expression [3,4]. Representing approximately 15–20% of all breast cancer cases, TNBC is characterized by limited therapeutic options, a high propensity for early metastasis, frequent recurrence, and poor clinical outcomes [5].

DTX, a taxane-based chemotherapeutic agent, is widely incorporated into treatment regimens for TNBC and several other solid malignancies. Its antitumor activity is primarily attributed to stabilization of microtubules, disruption of mitotic spindle dynamics, and induction of apoptotic cell death [6,7]. Despite its clinical effectiveness, the long-term therapeutic benefit of DTX is often compromised by dose-limiting toxicities, including neurotoxicity and hematological adverse effects, as well as the emergence of acquired drug resistance [8]. Consequently, increasing attention has been directed toward combination strategies that integrate conventional chemotherapeutic agents with naturally derived bioactive compounds to enhance therapeutic efficacy while potentially reducing toxicity and resistance development [9,10,11].

TQ (TQ; 2-isopropyl-5-methyl-1,4-benzoquinone), the principal bioactive constituent of *Nigella sativa* L. seed oil, has attracted considerable interest because of its broad spectrum of biological activities [12]. Previous studies have demonstrated antioxidant, anti-inflammatory, and anticancer properties of TQ across various experimental models [13,14,15]. In cancer cells, TQ has been reported to suppress proliferation, induce apoptosis, interfere with cell-cycle progression, inhibit angiogenesis, and attenuate metastatic behavior [16,17]. Mechanistically, these effects have been associated with modulation of PI3K/AKT/mTOR and MAPK signaling pathways, regulation of BCL-2 family proteins, disruption of mitochondrial integrity, and activation of apoptosis-related signaling networks [18,19]. Collectively, these observations suggest that TQ may represent a promising candidate for combination-based anticancer approaches.

ROS are key regulators of cellular homeostasis and participate in numerous signaling processes involved in cell survival, proliferation, and stress responses. Although physiological ROS levels are required for normal cellular function, excessive ROS accumulation can trigger oxidative stress, DNA damage, mitochondrial dysfunction, and apoptotic cell death [20]. Notably, several anticancer agents exert part of their cytotoxic activity through ROS generation and oxidative injury [21]. NAC, a precursor of glutathione biosynthesis and a well-established antioxidant, is frequently used to investigate the contribution of ROS-dependent mechanisms in cancer cell death and therapeutic responses [22].

Among the key mediators of intrinsic apoptosis, caspase-9 functions as an initiator caspase activated following mitochondrial cytochrome c release and apoptosome assembly [23]. Because activation of caspase-9 represents an important step in mitochondrial apoptotic signaling, assessment of its expression and localization may provide valuable insights into cellular responses induced by anticancer treatments [24].

The HaCaT cell line is a spontaneously immortalized human keratinocyte model that retains several characteristics of non-malignant epithelial cells and is commonly used in toxicological and safety-related investigations [25]. Comparative assessment of treatment responses in malignant and non-malignant cell models may provide preliminary information regarding differential cellular sensitivity and the potential therapeutic window of novel anticancer combinations [26].

Despite accumulating evidence supporting the anticancer activity of TQ, its potential to enhance DTX efficacy in TNBC and the molecular mechanisms underlying their interaction remain insufficiently characterized. In particular, the contribution of ROS-associated cellular stress, mitochondrial apoptotic signaling, and survival pathways involving *PIK3CA*, *AKT1*, and *PTEN* to the biological effects of combined TQ + DTX treatment has not been comprehensively investigated. Based on the complementary mechanisms of action reported for these agents, we hypothesized that TQ would potentiate the anticancer activity of DTX by enhancing ROS-associated stress responses, promoting apoptotic signaling, reducing wound closure, and modulating key genes involved in apoptosis and PI3K/AKT pathway regulation. To our knowledge, no previous study has comprehensively evaluated the combined effects of TQ + DTX on ROS-associated apoptosis, NAC-mediated rescue responses, migratory behavior, transcriptional regulation of apoptosis- and PI3K/AKT-related genes, and bioinformatic pathway analysis within a single TNBC experimental model. Therefore, the present study aimed to systematically evaluate the effects of TQ and DTX, administered individually and in combination, in MDA-MB-231 TNBC cells and HaCaT keratinocytes. To address this objective, a multi-parametric experimental approach incorporating combination-index analysis, Annexin V/PI flow cytometry, flow cytometric and fluorescence-based DCFH-DA ROS analyses with NAC rescue experiments, caspase-9 immunocytochemistry, wound-healing assays, quantitative real-time PCR, and bioinformatic analyses were employed to characterize the biological responses associated with TQ + DTX combination treatment and to explore its potential translational relevance.

## 2. Results

### 2.1. Effects of TQ and DTX on Cell Viability and Synergistic Interaction

**Figure 1:**
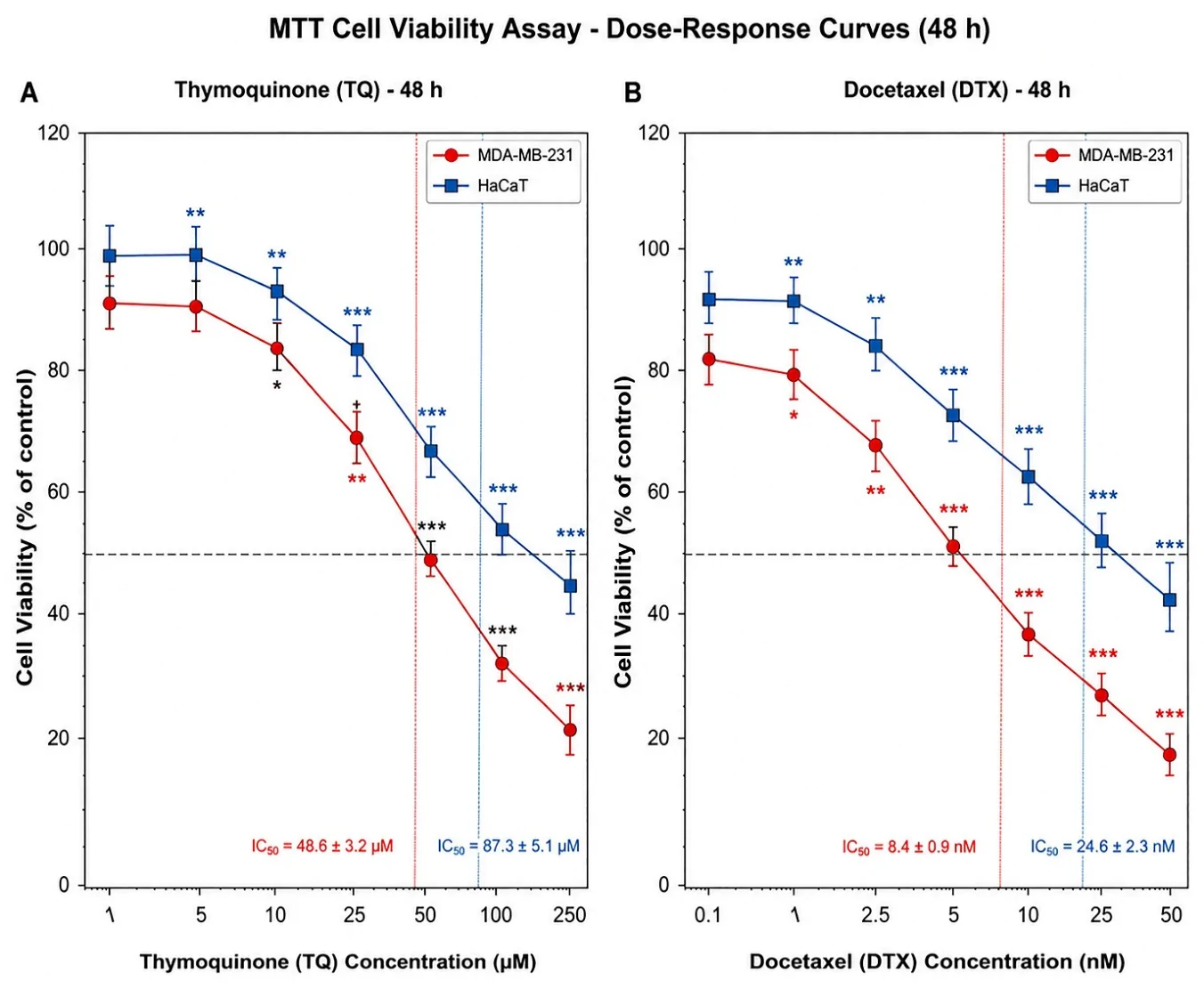
Dose-dependent cytotoxic effects of TQ and DTX in MDA-MB-231 and HaCaT cells following 48 h of treatment. (**A**) Cell viability of MDA-MB-231 and HaCaT cells following exposure to increasing concentrations of TQ (1–250 µM). (**B**) Cell viability of MDA-MB-231 and HaCaT cells following treatment with increasing concentrations of DTX (0.1–50 nM). IC_50_ values were determined by nonlinear regression analysis and are indicated on the graphs. MDA-MB-231 cells exhibited significantly lower IC_50_ values than HaCaT cells for both compounds, indicating greater sensitivity to TQ and DTX treatment. Data are presented as the mean ± SD of three independent biological experiments (*n* = 3). Statistical significance was determined using one-way ANOVA followed by Tukey’s multiple-comparison post hoc test. Red asterisks indicate comparisons with the untreated MDA-MB-231 control group, whereas blue asterisks indicate comparisons with the untreated HaCaT control group (* *p* < 0.05, ** *p* < 0.01, *** *p* < 0.001). IC_50_ values were calculated using GraphPad Prism version 9.0 (GraphPad Software, San Diego, CA, USA).

MTT assay results demonstrated dose-dependent reductions in cell viability following treatment with both TQ and DTX in MDA-MB-231 and HaCaT cells. In MDA-MB-231 cells, the IC_50_ values were determined as 48.6 ± 3.2 µM for TQ and 8.4 ± 0.9 nM for DTX. In HaCaT cells, the corresponding IC_50_ values were 87.3 ± 5.1 µM and 24.6 ± 2.3 nM, respectively (Figure 1). The higher IC_50_ values observed in HaCaT cells indicate lower sensitivity to both agents, suggesting preferential cytotoxic activity toward MDA-MB-231 cells under the present experimental conditions.

**Figure 2:**
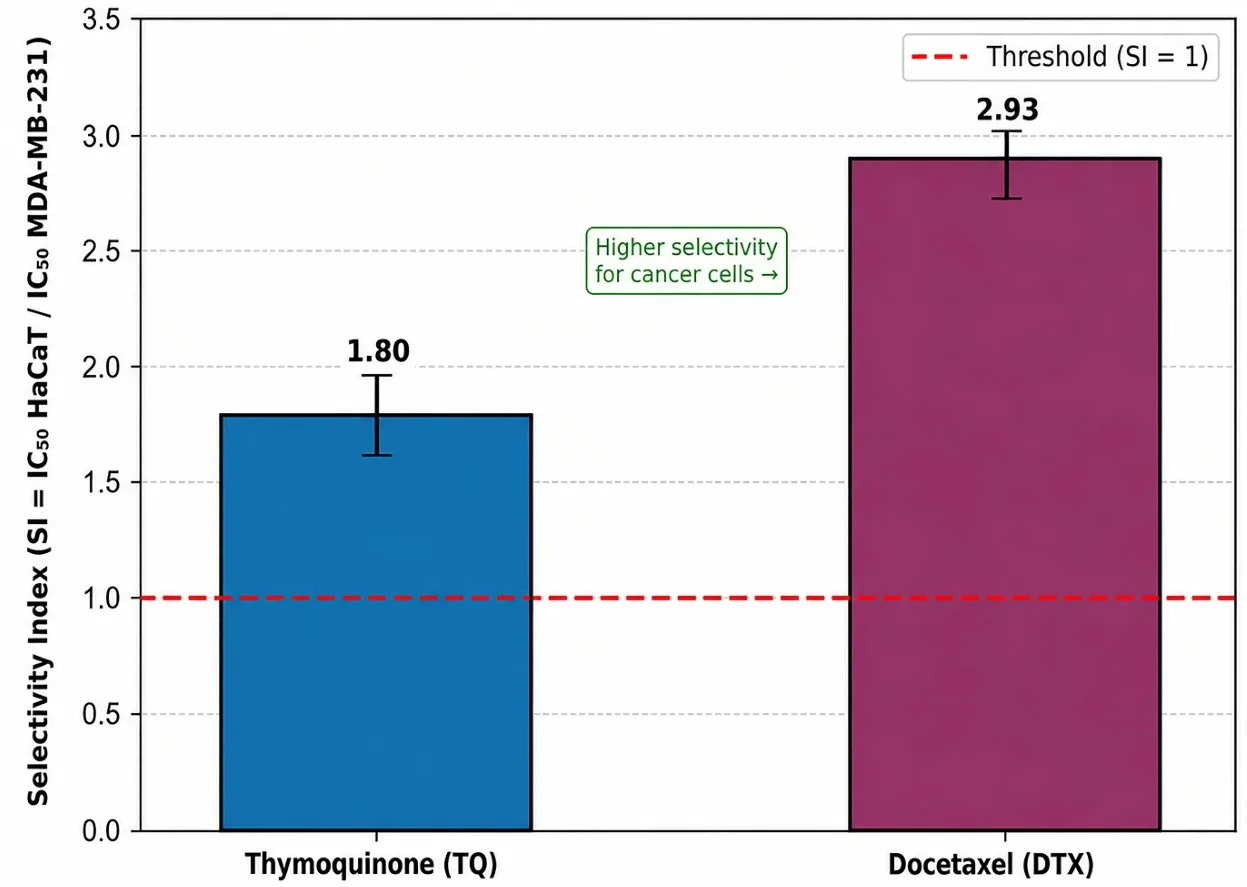
Selectivity indices (SI) of TQ and DTX in MDA-MB-231 and HaCaT cells. The SI was calculated as the ratio of the IC_50_ value in HaCaT cells to that in MDA-MB-231 cells (SI = IC_50_ HaCaT/IC_50_ MDA-MB-231). The calculated SI values were 1.80 for TQ and 2.93 for DTX, indicating preferential cytotoxicity toward MDA-MB-231 breast cancer cells relative to the non-malignant HaCaT cell line. The dashed red line represents the reference threshold (SI = 1), with SI values >1 indicating selective activity against cancer cells. Data are presented as the mean ± SD of IC_50_ values obtained from three independent biological experiments (*n* = 3).

Selectivity index (SI) analysis further supported these observations. The calculated SI values were 1.80 for TQ and 2.93 for DTX, indicating greater cytotoxic activity toward MDA-MB-231 cells than toward HaCaT cells (Figure 2). However, these findings should be interpreted as evidence of differential cellular sensitivity between the malignant and non-malignant cell models evaluated in the present study.

**Figure 3:**
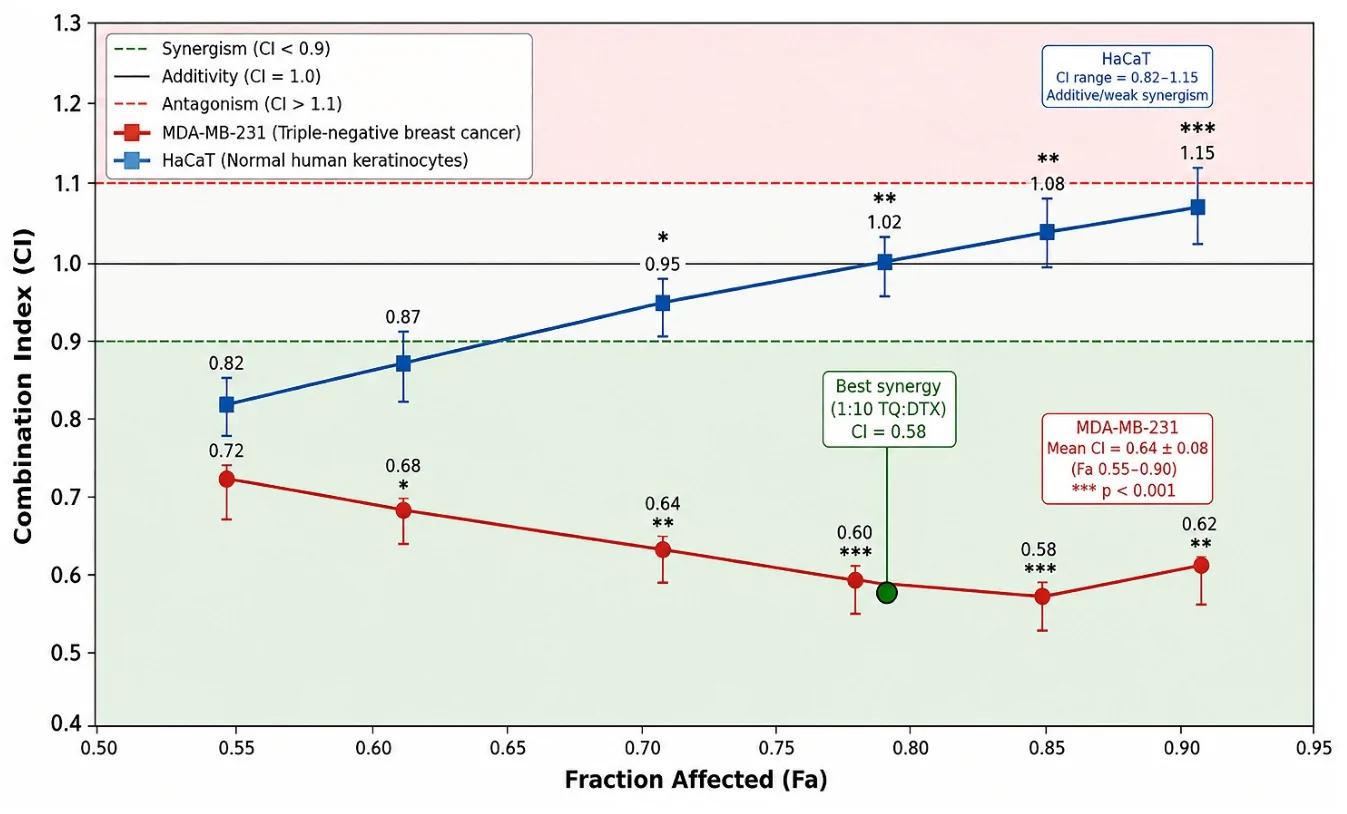
CI analysis of TQ and DTX in MDA-MB-231 and HaCaT cells. CI values were calculated using CompuSyn software (version 1.0; ComboSyn Inc., Paramus, NJ, USA). according to the Chou–Talalay method and plotted against the fraction affected (Fa). CI values < 0.90 indicate synergism, values between 0.90 and 1.10 indicate additive interactions, and values > 1.10 indicate antagonism. In MDA-MB-231 cells, CI values ranged from 0.58 to 0.72, with a mean CI of 0.64 ± 0.08 across the evaluated Fa range (0.55–0.90), demonstrating consistent synergistic interactions. The strongest synergistic effect was observed at Fa = 0.85 (CI = 0.58). In contrast, HaCaT cells exhibited CI values ranging from 0.82 to 1.15, corresponding predominantly to additive or weakly synergistic interactions. Data are presented as the mean ± SD of three independent biological experiments (*n* = 3). * *p* < 0.05, ** *p* < 0.01, and *** *p* < 0.001.

CI analysis performed using the Chou–Talalay method demonstrated synergistic interactions between TQ and DTX in MDA-MB-231 cells across the evaluated fraction affected (Fa) range. CI values remained below 0.90 throughout the tested Fa interval (0.55–0.90), with a mean CI value of 0.64 ± 0.08, indicating consistent synergism (Figure 3). The strongest synergistic interaction was observed at Fa ≈ 0.85, where the CI value reached 0.58. In contrast, CI values in HaCaT cells ranged from 0.82 to 1.15, indicating a transition from weakly synergistic to additive and mildly antagonistic interactions at higher Fa levels. These findings support the presence of pharmacological synergism between TQ and DTX specifically in TNBC cells while demonstrating comparatively weaker interactions in non-malignant HaCaT cells.

### 2.2. Apoptotic Cell Death Induced by TQ and DTX in MDA-MB-231 Cells

**Figure 4:**
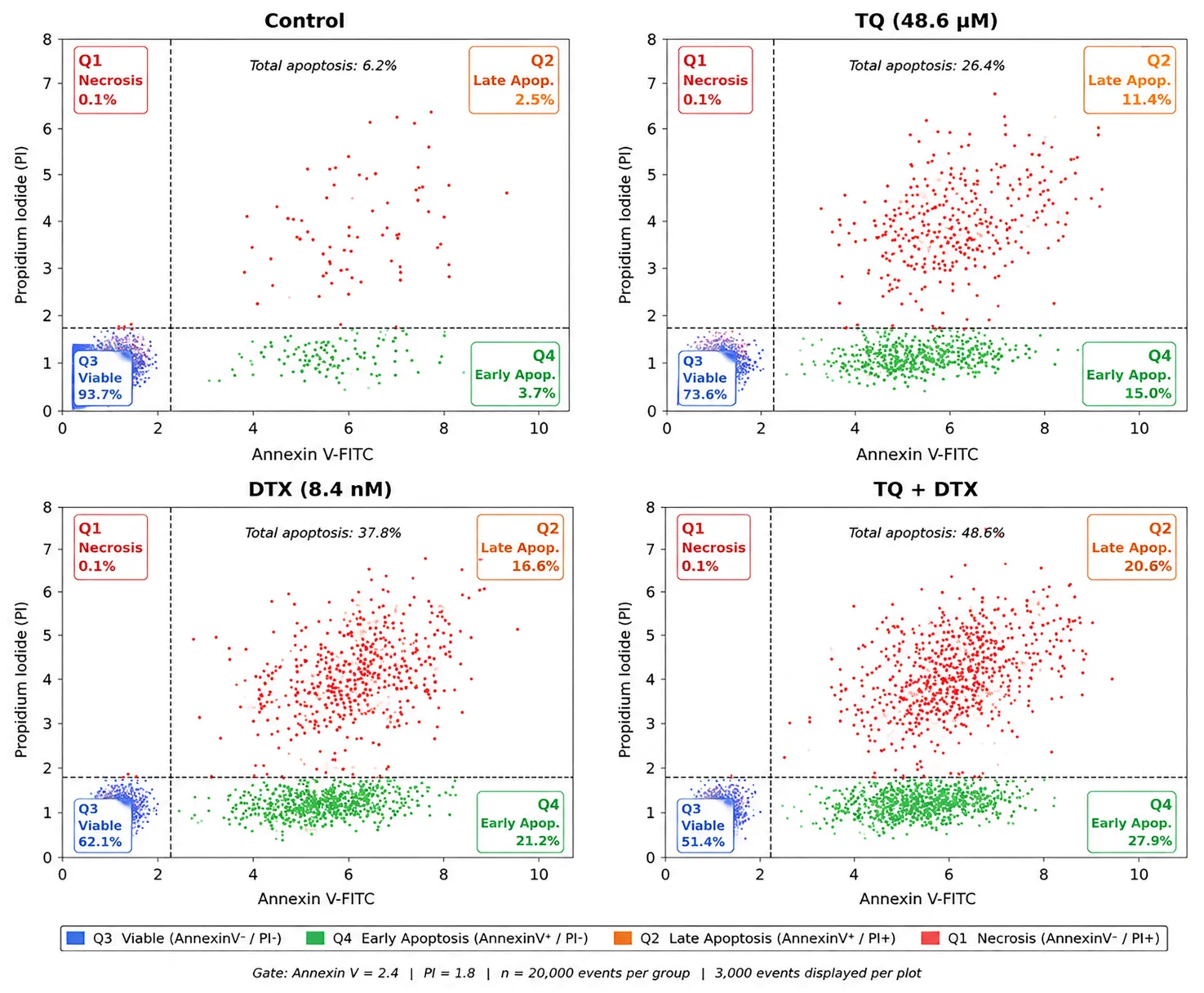
Representative Annexin V-FITC/PI flow cytometric analysis of apoptosis in MDA-MB-231 cells following treatment with TQ, DTX, and their combination. Cells were treated with TQ (48.6 µM), DTX (8.4 nM), or the TQ + DTX combination for 48 h and subsequently analyzed by Annexin V-FITC/PI staining. Quadrants represent viable cells (Annexin V^−^/PI^−^; Q3), early apoptotic cells (Annexin V^+^/PI^−^; Q4), late apoptotic cells (Annexin V^+^/PI^+^; Q2), and necrotic cells (Annexin V^−^/PI^+^; Q1). Total apoptosis was calculated as the sum of early and late apoptotic populations. The TQ + DTX combination produced the highest apoptotic response (48.6%), compared with TQ (26.4%), DTX (37.8%), and untreated control cells (6.2%). Representative dot plots from three independent biological experiments are shown.

Flow cytometric analysis using Annexin V-FITC/propidium iodide (PI) double staining was performed to quantitatively evaluate apoptosis in MDA-MB-231 cells following 48 h of treatment with TQ, DTX, and their combination (Figure 4). The assay enabled discrimination of viable (Annexin V^−^/PI^−^), early apoptotic (Annexin V^+^/PI^−^), late apoptotic (Annexin V^+^/PI^+^), and necrotic (Annexin V^−^/PI^+^) cell populations.

In untreated control cells, total apoptosis was 6.2 ± 0.8%, consisting of 3.7 ± 0.5% early apoptotic and 2.5 ± 0.4% late apoptotic cells, indicating a low basal rate of spontaneous cell death.

Treatment with TQ (48.6 µM) significantly increased total apoptosis to 26.4 ± 1.9% (*p* < 0.001 vs. control), comprising 15.0 ± 1.4% early apoptotic and 11.4 ± 1.1% late apoptotic cells.

DTX treatment (8.4 nM) induced a more pronounced apoptotic response, increasing total apoptosis to 37.8 ± 2.1% (*p* < 0.001 vs. control; *p* < 0.01 vs. TQ). Early and late apoptotic populations accounted for 21.2 ± 1.6% and 16.6 ± 1.3% of cells, respectively.

The TQ + DTX combination produced the strongest apoptotic response among all treatment groups. Total apoptosis reached 48.6 ± 3.7%, representing a significant increase compared with both TQ and DTX monotherapies (*p* < 0.001). Early and late apoptotic populations were 27.9 ± 2.4% and 20.6 ± 1.8%, respectively, indicating that combined treatment potentiated apoptosis beyond the effects achieved by either monotherapy.

### 2.3. Effects of TQ and DTX on Fluorescent Cell Density Assessed by TALI Cytometry

**Figure 5:**
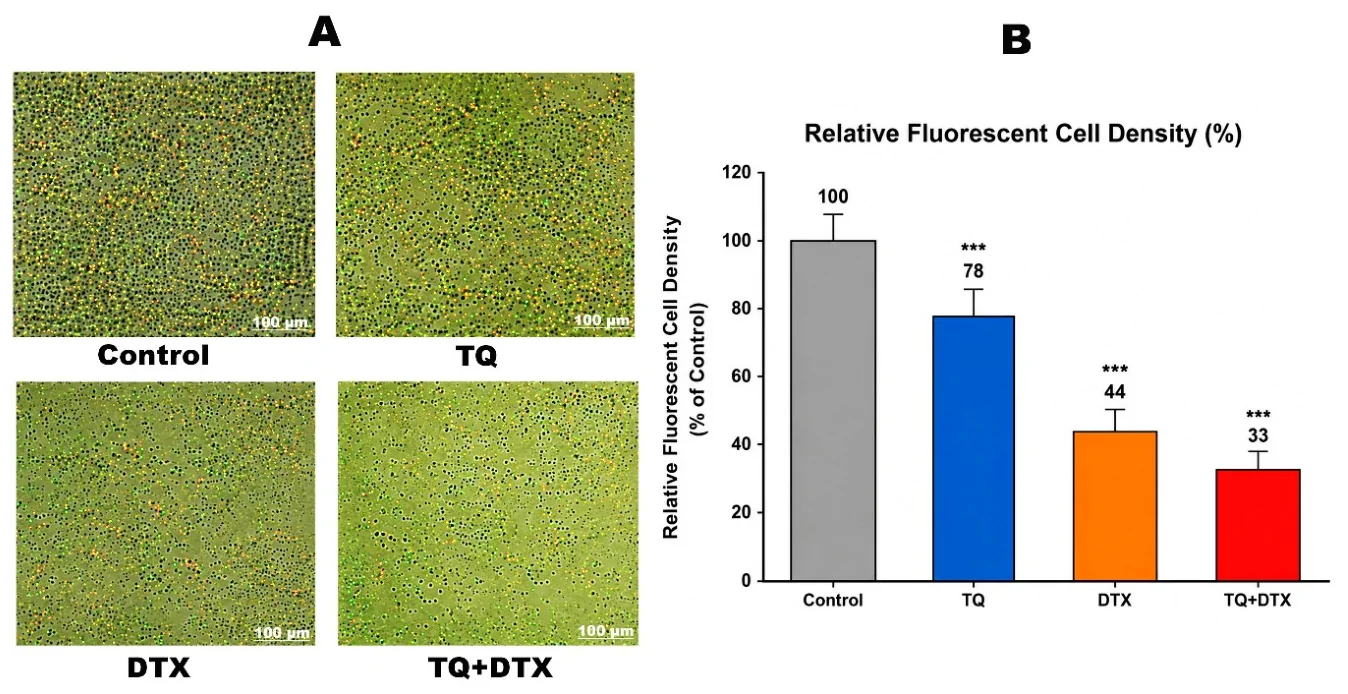
TALI image-based cytometric analysis of MDA-MB-231 cells following treatment with TQ, DTX, and their combination. (**A**) Representative fluorescence images acquired after 48 h of treatment using the TALI^®^ Image-Based Cytometer. (**B**) Quantitative analysis of relative fluorescent cell density normalized to the untreated control group. Treatment with TQ or DTX reduced fluorescent cell density compared with the control, whereas the TQ + DTX combination produced the greatest reduction, indicating enhanced cytotoxic activity. Data are presented as the mean ± SD of three independent biological experiments (*n* = 3). Statistical significance was determined by one-way ANOVA followed by Tukey’s multiple comparisons post hoc test. *** *p* < 0.001 versus the untreated control group.

The effects of TQ and DTX on MDA-MB-231 cells were further evaluated using a TALI image-based cytometry system with dual-fluorescence staining (Figure 5). Representative fluorescence images demonstrated a progressive reduction in fluorescently labeled cell density following treatment with TQ, DTX, and their combination compared with untreated control cells (Figure 5A).

Quantitative analysis confirmed these observations (Figure 5B). Relative to the control group (100%), fluorescent cell density decreased to 78% following treatment with TQ (48.6 µM). A more pronounced reduction was observed in DTX-treated cells (8.4 nM), where fluorescent cell density declined to 44% of the control level. The greatest reduction was detected in the TQ + DTX combination group, in which fluorescent cell density decreased to 33% of the control value (*p* < 0.001 vs. control). Overall, the combination treatment produced the most substantial decrease in fluorescently labeled cell density, consistent with the enhanced cytotoxic and pro-apoptotic effects observed in the MTT and Annexin V/PI analyses. These findings further support the enhanced antiproliferative activity of the combination treatment observed in the MTT assay.

### 2.4. ROS Production and NAC Rescue Experiment

**Figure 6:**
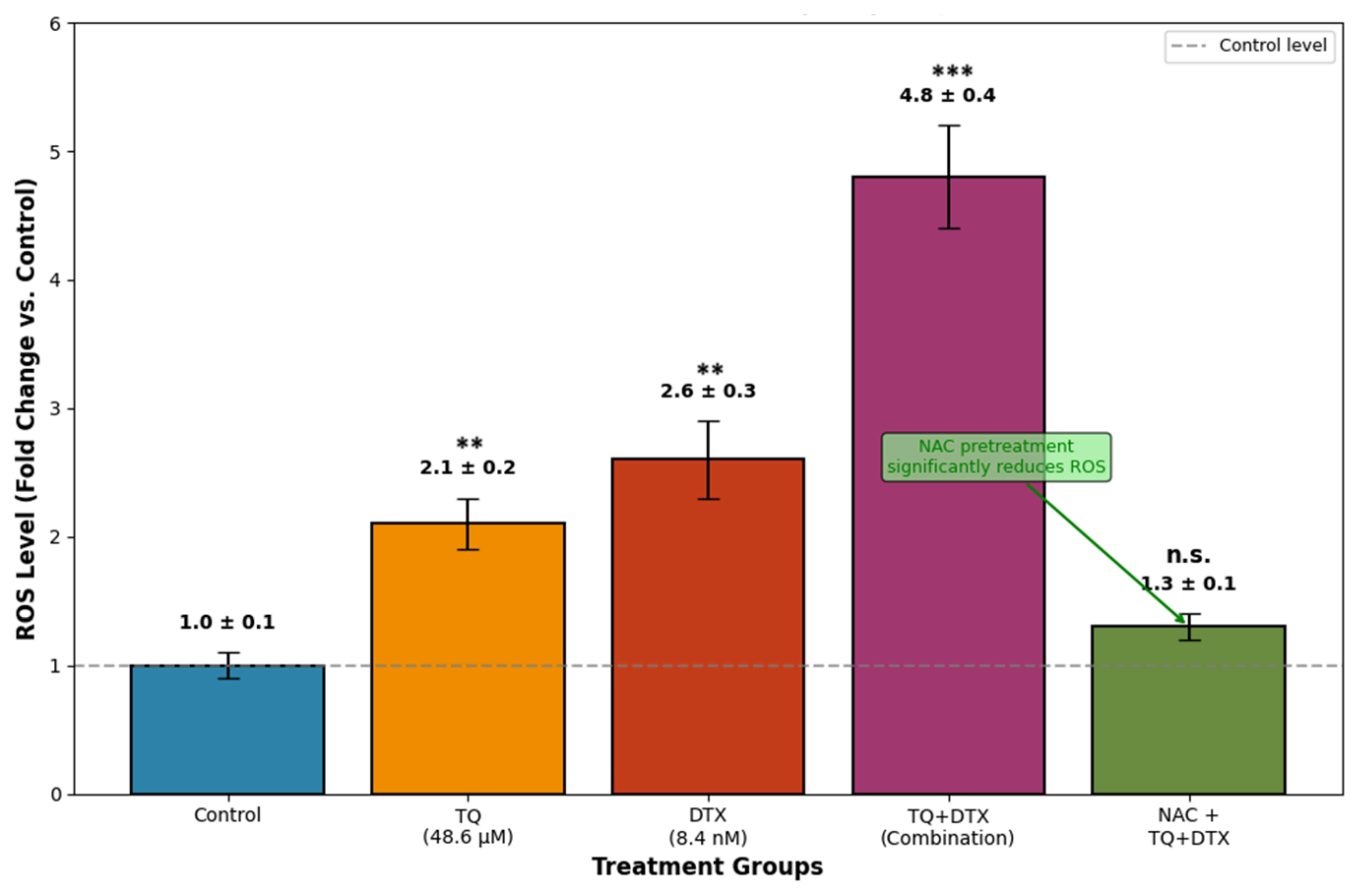
Intracellular ROS production and NAC rescue analysis in MDA-MB-231 cells. Intracellular ROS levels were determined by DCFH-DA fluorescence following 48 h treatment with TQ (TQ, 48.6 µM), DTX (DTX, 8.4 nM), and their combination (TQ + DTX). The combination treatment induced the highest ROS accumulation (4.8 ± 0.4-fold relative to control), whereas NAC pretreatment (5 mM, 1 h) markedly attenuated ROS generation, reducing ROS levels, although they remained slightly above those of untreated control cells. Data are presented as mean ± SD from three independent experiments. Statistical significance was determined by one-way ANOVA followed by Tukey’s post hoc test. ** *p* < 0.01 and *** *p* < 0.001 versus control; n.s., not significant versus control. Representative histograms are shown together with quantitative analyses.

Intracellular ROS levels were evaluated using DCFH-DA fluorescence staining following treatment with TQ, DTX, and their combination in MDA-MB-231 cells (Figure 6). Compared with untreated control cells, TQ treatment increased ROS levels to 2.1 ± 0.2-fold, whereas DTX treatment increased ROS levels to 2.6 ± 0.3-fold relative to control. The TQ + DTX combination induced the highest ROS accumulation, reaching 4.8 ± 0.4-fold above the control level (*p* < 0.001). NAC pretreatment (5 mM, 1 h) markedly attenuated ROS generation, reducing ROS levels in the combination group to 1.3 ± 0.1-fold relative to control.

To further investigate the contribution of ROS to treatment-induced cytotoxicity, an NAC rescue experiment was performed. NAC pretreatment increased cell viability from 54.6% to 81.2% and reduced the apoptosis rate from 48.6% to 18.3% in cells treated with the TQ + DTX combination (*p* < 0.001). These findings indicate that suppression of ROS generation partially reversed the cytotoxic and pro-apoptotic effects induced by the combination treatment.

**Figure 7:**
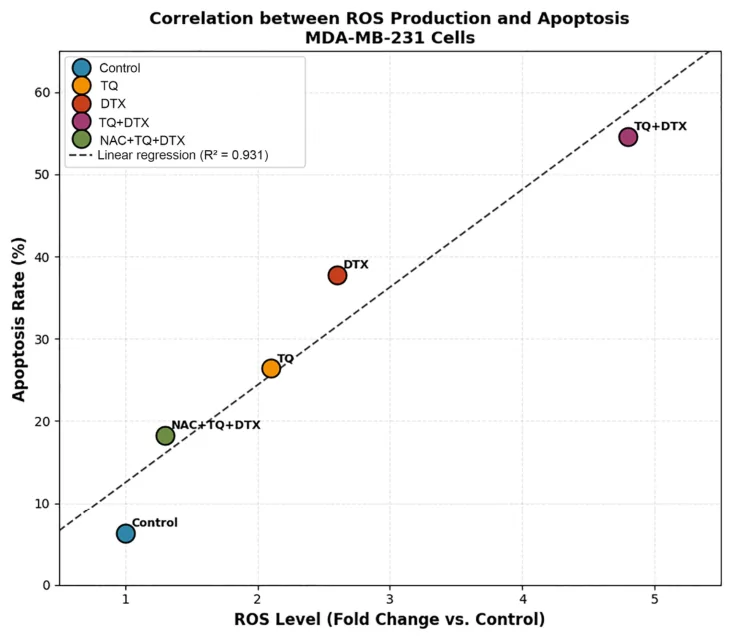
Correlation between intracellular ROS levels and apoptosis rates in MDA-MB-231 cells. Scatter plot illustrating the relationship between the mean intracellular ROS levels and the corresponding mean total apoptosis rates across the experimental groups, including Control, TQ, DTX, TQ + DTX combination, and NAC-pretreated TQ + DTX cells. The plot illustrates the overall trend between the mean ROS and mean apoptosis values across the experimental groups. The dashed line represents the linear regression fit. Each data point corresponds to the mean value of three independent biological experiments for the respective experimental group.

Correlation analysis demonstrated a strong positive association between intracellular ROS levels and apoptosis rates across the experimental groups (R^2^ = 0.931, *p* < 0.001; Figure 7). Collectively, these findings suggest that ROS contributes to the cytotoxic and apoptotic responses observed following TQ + DTX treatment in MDA-MB-231 cells. The marked reduction in ROS levels following NAC pretreatment further supports the involvement of ROS in the cytotoxic effects induced by the TQ + DTX combination.

### 2.5. Fluorescence Visualization of Intracellular ROS Accumulation in MDA-MB-231 Cells

**Figure 8:**
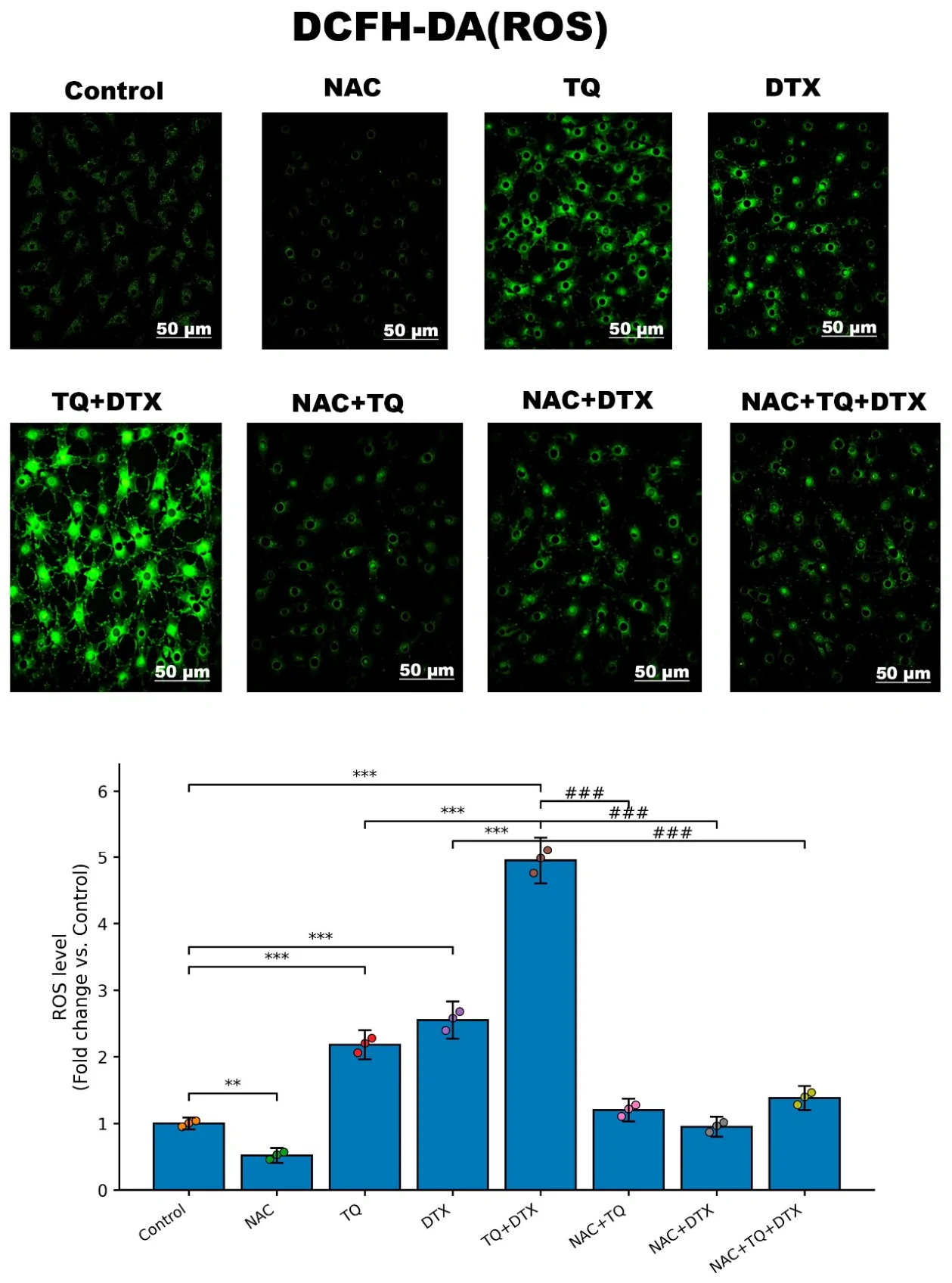
Representative DCFH-DA fluorescence images and quantitative analysis of intracellular ROS levels in MDA-MB-231 cells following treatment with TQ, DTX, their combination (TQ + DTX), and the corresponding N-acetyl-L-cysteine (NAC)-pretreated groups. Representative fluorescence images (**upper panels**) demonstrate intracellular ROS accumulation after 48 h of treatment. Quantitative fluorescence analysis (**lower panel**) shows intracellular ROS levels expressed as a fold change relative to the untreated control group. Data are presented as mean ± SD from three independent experiments (*n* = 3). Statistical significance was determined by one-way ANOVA followed by Tukey’s multiple-comparison test. ** *p* < 0.01, *** *p* < 0.001 versus Control; ### *p* < 0.001 versus TQ + DTX. Scale bar = 50 μm.

Representative DCFH-DA fluorescence images demonstrated low basal intracellular ROS-associated fluorescence in control and NAC-treated MDA-MB-231 cells (Figure 8). In contrast, treatment with either TQ or DTX markedly increased green fluorescence intensity, indicating enhanced intracellular ROS generation. The TQ + DTX combination produced the strongest fluorescence signal throughout the cytoplasm, reflecting substantially greater intracellular ROS accumulation than either monotherapy. Pretreatment with NAC markedly reduced fluorescence intensity in the TQ-, DTX-, and particularly the TQ + DTX-treated groups, although weak residual fluorescence remained detectable following combination treatment. Importantly, the fluorescence staining pattern closely paralleled the flow cytometric ROS findings, supporting the reproducibility of treatment-associated oxidative stress across two independent analytical approaches.

Semi-quantitative fluorescence intensity analysis further supported these qualitative observations (Figure 8). Compared with the control group, TQ and DTX significantly increased intracellular ROS levels (both *** *p* < 0.001), whereas the TQ + DTX combination induced the highest ROS accumulation, reaching approximately 5-fold above control (*** *p* < 0.001). NAC pretreatment significantly reduced ROS production in TQ-, DTX-, and combination-treated cells (*** *p* < 0.001), demonstrating effective suppression of treatment-induced oxidative stress. Nevertheless, ROS levels in the NAC + TQ + DTX group remained modestly elevated compared with untreated control cells, indicating that NAC only partially reversed the oxidative response induced by the combination treatment.

Collectively, these fluorescence imaging and quantitative analyses confirm that the TQ + DTX combination markedly potentiates intracellular ROS accumulation in MDA-MB-231 cells, while NAC pretreatment substantially—but not completely—attenuates treatment-associated oxidative stress. These findings are consistent with a contribution of ROS to the observed antitumor effects of the combined treatment.

### 2.6. Immunocytochemical Staining Findings

**Figure 9:**
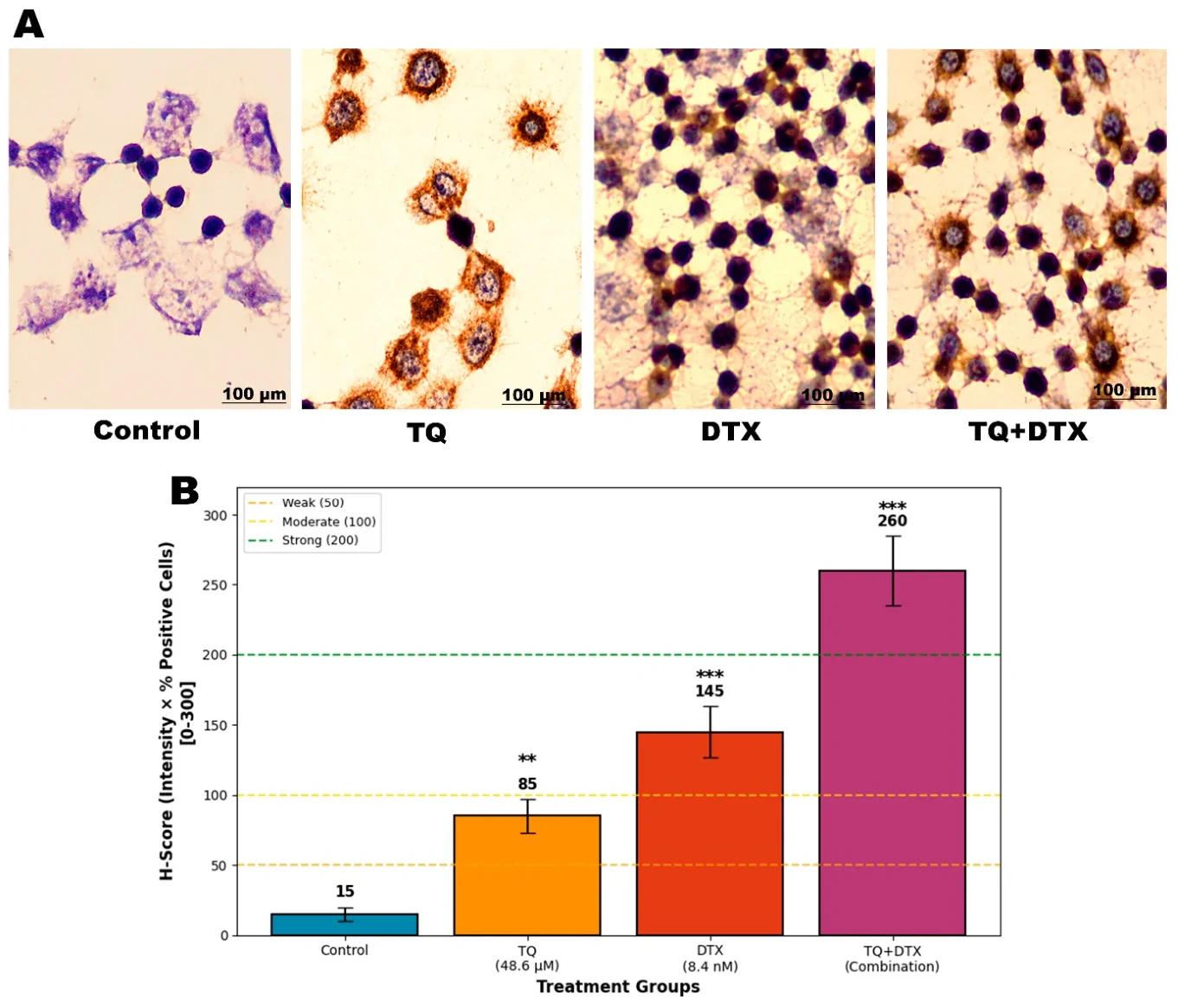
Immunocytochemical evaluation of caspase-9 expression in MDA-MB-231 cells following treatment with TQ, DTX, and their combination. (**A**) Representative immunocytochemical images showing caspase-9 immunoreactivity. Brown DAB staining indicates positive caspase-9 expression, while nuclei were counterstained with hematoxylin. Scale bar = 100 µm. (**B**) Quantitative H-score analysis of caspase-9 staining intensity. H-scores were calculated using the formula H-score = Σ(Pi × i), with values ranging from 0 to 300. Dashed lines indicate weak (50), moderate (100), and strong (200) staining thresholds. Data are presented as mean ± SD from three independent experiments. ** *p* < 0.01 and *** *p* < 0.001 versus control. Representative images from three independent experiments are shown.

Immunocytochemical staining revealed a treatment-dependent increase in caspase-9 immunoreactivity in MDA-MB-231 cells (Figure 9A). Untreated control cells exhibited minimal DAB staining with weak cytoplasmic immunoreactivity and preserved cellular morphology, yielding an H-score of 15, indicative of minimal caspase-9 immunoreactivity. Treatment with TQ (48.6 µM) increased cytoplasmic DAB staining intensity, resulting in an H-score of 85 (*p* < 0.01 vs. control), corresponding to a weak-to-moderate staining pattern. DTX treatment (8.4 nM) produced a more pronounced immunoreactive signal, with an H-score of 145 (*p* < 0.001 vs. control), consistent with moderate-to-strong caspase-9 expression. In addition, DTX-treated cells exhibited morphological alterations including reduced cellular spreading and increased cellular condensation. The strongest caspase-9 immunoreactivity was observed in the TQ + DTX combination group, which achieved an H-score of 260 (*p* < 0.001 vs. control), corresponding to strong staining intensity. Representative images demonstrated extensive cytoplasmic DAB positivity accompanied by marked morphological alterations. Quantitative H-score analysis confirmed that combination treatment produced the highest level of caspase-9 immunoreactivity among all experimental groups (Figure 9B).

### 2.7. TQ and DTX Suppress Wound Closure in MDA-MB-231 Cells

**Figure 10:**
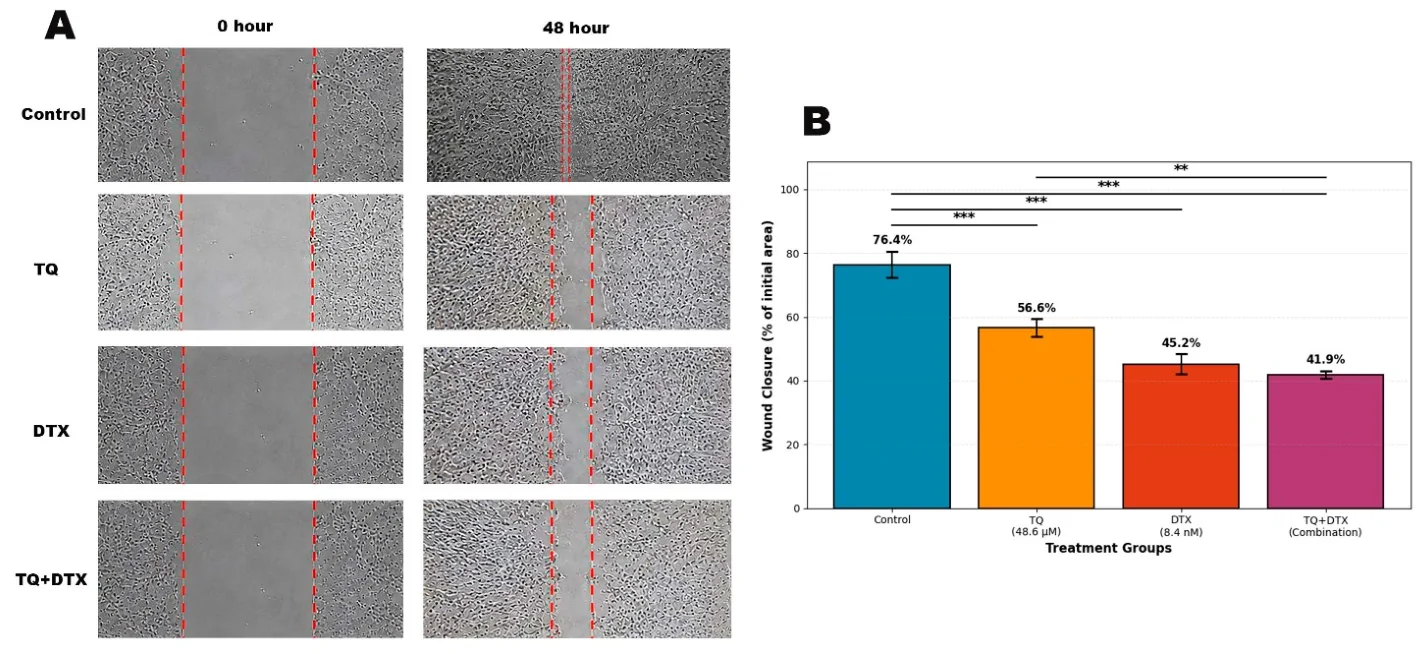
Effect of TQ and DTX on the migratory capacity of MDA-MB-231 cells assessed by wound healing assay. (**A**) Representative images of scratch wounds immediately after scratching (0 h) and after 48 h of treatment with TQ (48.6 µM), DTX (8.4 nM), or their combination (TQ + DTX). A linear scratch wound was generated using a sterile 200 µL pipette tip and images were acquired with an inverted microscope (scale bar = 100 µm). The red dashed lines indicate the wound edges at 0 h and the remaining wound boundaries at 48 h. (**B**) Quantitative analysis of wound closure. Wound closure was quantified using ImageJ software (version 1.54; National Institutes of Health, Bethesda, MD, USA) and calculated as [(initial wound area − final wound area)/initial wound area] × 100. Data are presented as mean ± SD (*n* = 3). Wound closure percentages were 76.4 ± 4.2% (Control), 56.6 ± 2.8% (TQ), 45.2 ± 3.1% (DTX), and 41.9 ± 1.2% (TQ + DTX). Statistical significance was determined by one-way ANOVA followed by Tukey’s multiple comparison test. ** *p* < 0.01, *** *p* < 0.001.

Representative wound healing images and quantitative analysis are presented in Figure 10. After 48 h, untreated control cells exhibited 76.4 ± 4.2% wound closure, indicating extensive wound closure. Treatment with TQ significantly reduced wound closure to 56.6 ± 2.8% (*p* < 0.001 vs. control), whereas DTX further decreased wound closure to 45.2 ± 3.1% (*** *p* < 0.001 vs. control). The TQ + DTX combination produced the lowest wound closure (41.9 ± 1.2%), corresponding to the greatest suppression of wound closure among all treatment groups (*** *p* < 0.001 vs. control). Quantitative comparisons further demonstrated that wound closure in the combination group was significantly lower than that observed following TQ treatment (*** *p* < 0.001) and remained modestly but significantly lower than that following DTX treatment (** *p* < 0.01). Overall, these findings indicate that the combined treatment was associated with a greater reduction in wound closure than either monotherapy (Figure 10).

### 2.8. Effects of TQ and DTX on Apoptosis- and PI3K/AKT Pathway-Related Gene Expression

**Figure 11:**
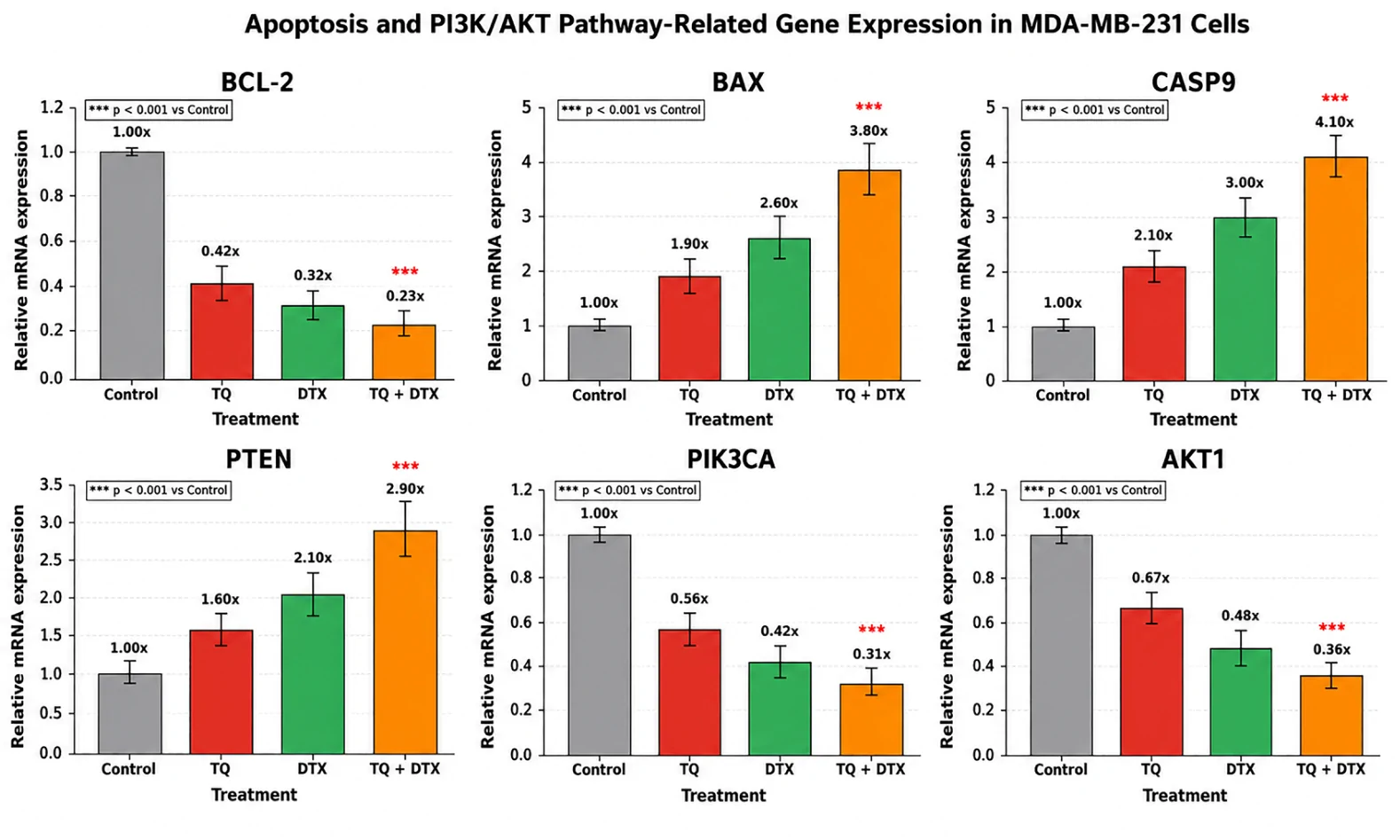
Relative mRNA expression of apoptosis- and PI3K/AKT pathway-related genes in MDA-MB-231 cells following treatment with TQ (TQ, 48.6 µM), DTX (DTX, 8.4 nM), or their combination for 48 h. Relative expression levels of the apoptosis-related genes *BCL2*, *BAX*, and *CASP9*, together with the PI3K/AKT pathway-related genes *PTEN*, *PIK3CA*, and *AKT1*, were determined by quantitative real-time PCR (qRT-PCR). Gene expression levels are expressed as a fold change relative to the untreated control group (set to 1.0). Data are presented as mean ± SD (*n* = 3). Statistical significance is indicated in the corresponding panels. *** *p* < 0.001 versus the untreated control group.

Quantitative real-time PCR (qRT-PCR) was performed to evaluate the mRNA expression levels of the apoptosis-related genes *BCL2*, *BAX*, and *CASP9*, together with the PI3K/AKT pathway-associated genes *PTEN*, *PIK3CA*, and *AKT1,* in MDA-MB-231 cells following 48 h of treatment with TQ, DTX, or their combination (Figure 11).

Expression of the anti-apoptotic gene *BCL2* was progressively reduced in all treatment groups, reaching 0.42-fold, 0.32-fold, and 0.23-fold of control levels following TQ, DTX, and TQ + DTX treatment, respectively (*** *p* < 0.001). In contrast, the pro-apoptotic genes *BAX* and *CASP9* were upregulated in a treatment-dependent manner. *BAX* expression increased to 1.90-fold, 2.60-fold, and 3.80-fold, whereas *CASP9* expression increased to 2.10-fold, 3.00-fold, and 4.10-fold following TQ, DTX, and combination treatment, respectively (*** *p* < 0.001).

**Figure 12:**
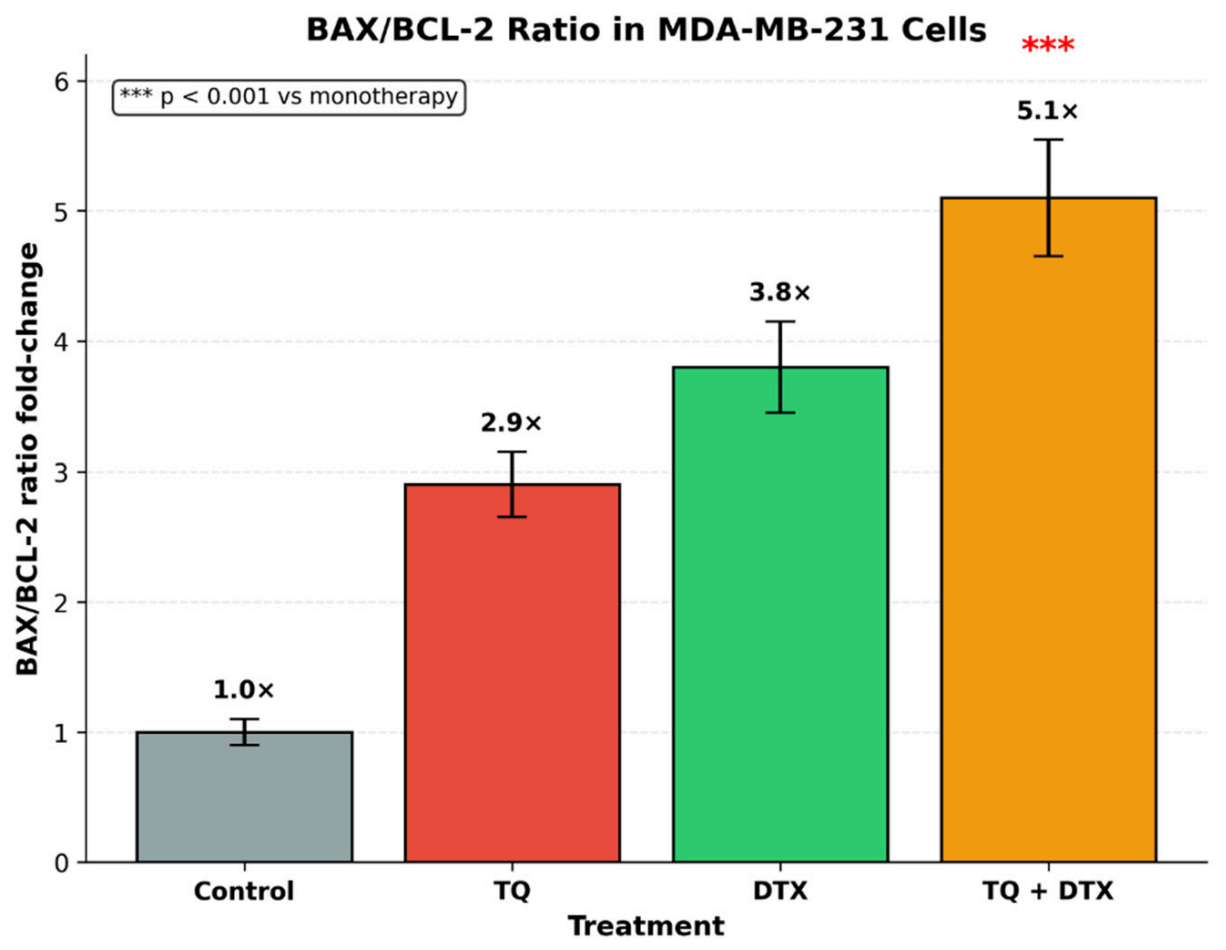
Relative *BAX/BCL2* expression ratio in MDA-MB-231 cells following treatment with TQ (TQ, 48.6 µM), DTX (DTX, 8.4 nM), or their combination for 48 h. The BAX/BCL2 ratio was calculated for each independent biological replicate using the normalized relative expression (2^−ΔΔCt^) values of *BAX* and *BCL2* and is presented as a fold change relative to the untreated control group (set to 1.0). Data are presented as mean ± SD (*n* = 3). *** *p* < 0.001 versus monotherapy groups (one-way ANOVA followed by Tukey’s multiple comparison test).

To further evaluate the balance between pro- and anti-apoptotic signaling, the BAX/BCL2 expression ratio was calculated for each independent biological replicate using the normalized relative expression (2^−ΔΔCt^) values of *BAX* and *BCL2*, as described in the Methods (Figure 12). The ratio increased from 1.0-fold in control cells to 2.9-fold, 3.8-fold, and 5.1-fold following TQ, DTX, and TQ + DTX treatment, respectively, with the combination group exhibiting the highest ratio (*** *p* < 0.001).

Analysis of PI3K/AKT pathway-related genes demonstrated a treatment-dependent increase in *PTEN* expression together with reduced expression of *PIK3CA* and *AKT1*. *PTEN* expression increased to 1.60-fold, 2.10-fold, and 2.90-fold, whereas *PIK3CA* expression decreased to 0.56-fold, 0.42-fold, and 0.31-fold of control levels following TQ, DTX, and combination treatment, respectively. Similarly, *AKT1* expression decreased to 0.67-fold, 0.48-fold, and 0.36-fold of control levels, with the greatest changes consistently observed in the combination group (*** *p* < 0.001) (Figure 11).

### 2.9. Bioinformatics Findings

**Figure 13:**
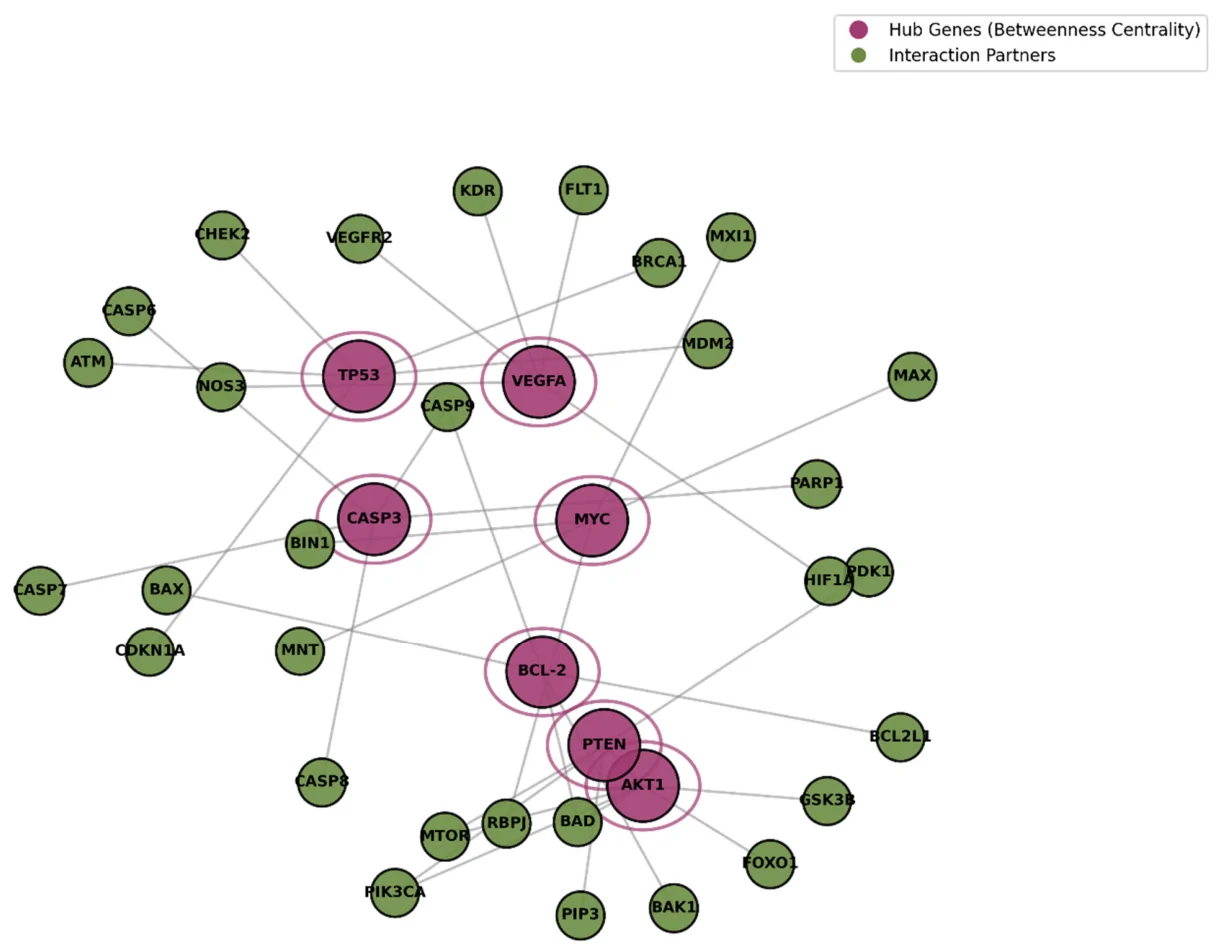
STRING PPI network analysis. The network comprises 68 nodes and 341 edges. Hub genes, identified based on betweenness centrality analysis, are highlighted in purple and include *TP53*, *AKT1*, *BCL2*, *CASP3*, *PTEN*, *MYC*, and *VEGFA*. Green nodes represent interacting proteins, whereas edges indicate known or predicted protein–protein interactions. Larger node size indicates hub genes with higher betweenness centrality.

Protein–protein interaction (PPI) analysis performed using the STRING database generated an interaction network comprising 68 nodes and 341 edges. Based on betweenness centrality analysis, *TP53*, *AKT1*, *BCL2*, *CASP3*, *PTEN*, *MYC*, and *VEGFA* were identified as the principal hub genes within the interaction network (Figure 13).

**Figure 14:**
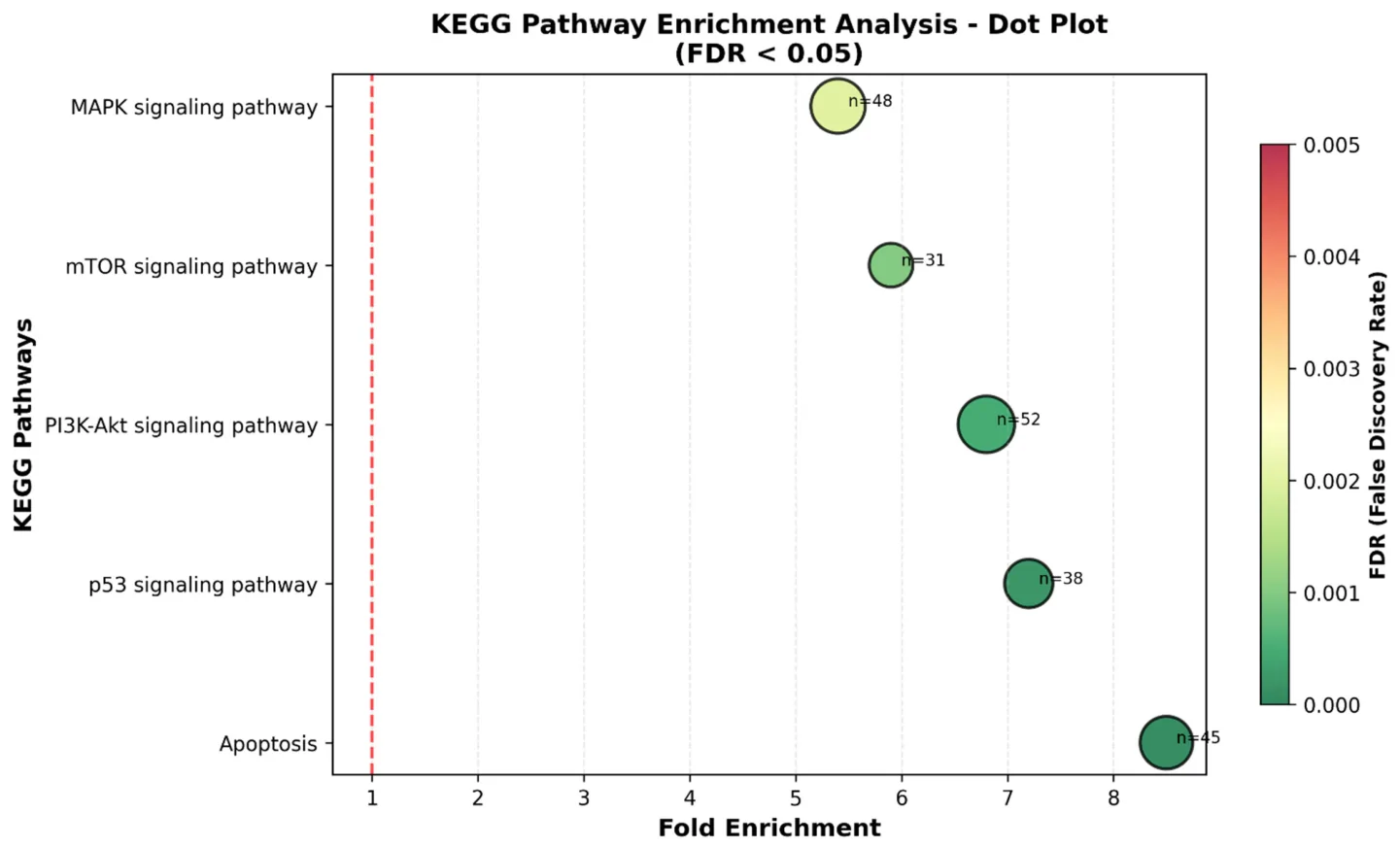
KEGG pathway enrichment analysis of the differentially expressed genes. The most significantly enriched pathways included apoptosis (hsa04210), p53 signaling (hsa04115), PI3K–Akt signaling (hsa04151), mTOR signaling (hsa04150), and MAPK signaling (hsa04010), with fold enrichment values of 8.5, 7.2, 6.8, 5.9, and 5.4, respectively. All enriched pathways met the significance threshold of FDR < 0.05.

**Figure 15:**
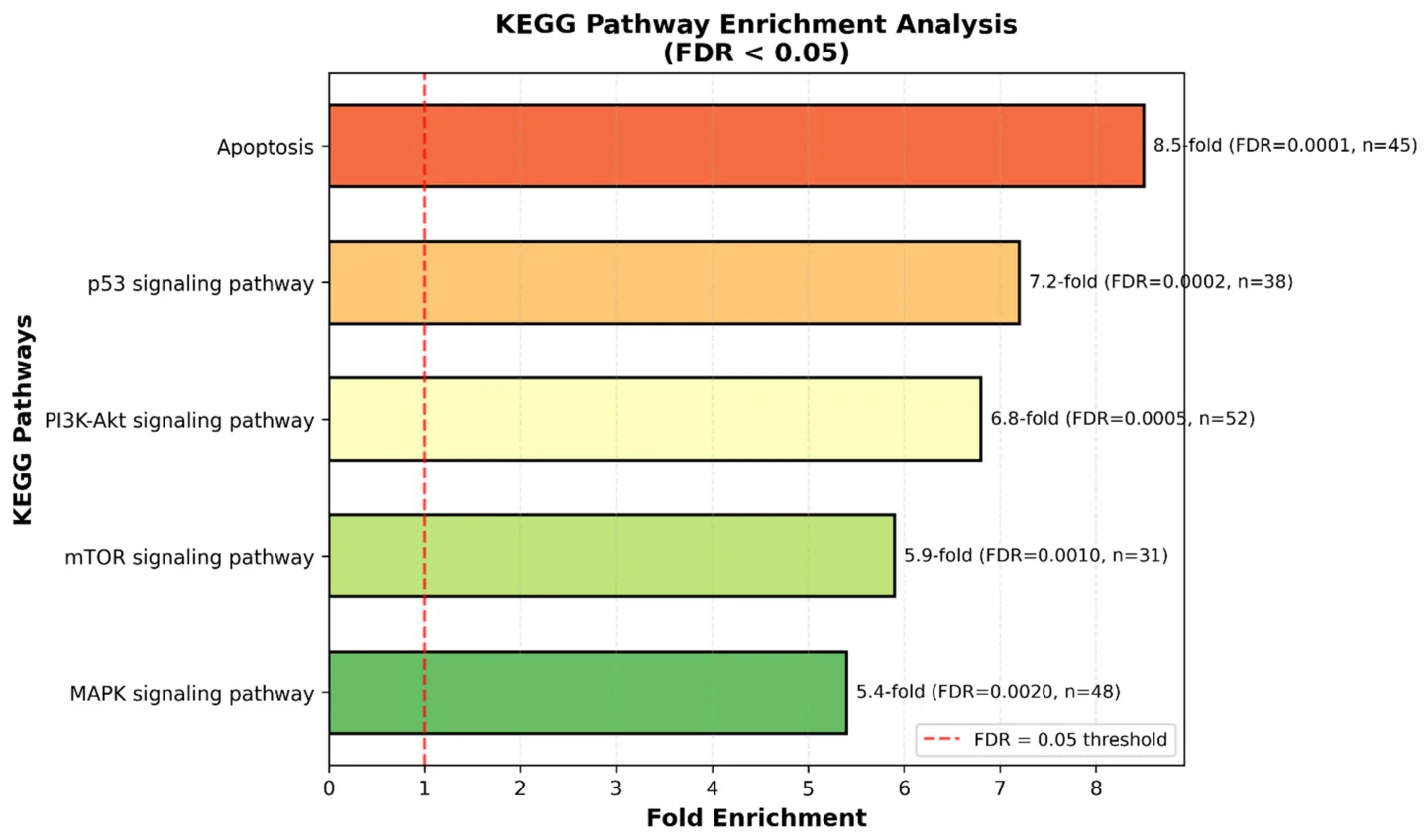
Bubble plot of KEGG pathway enrichment analysis. Apoptosis, p53 signaling, PI3K–Akt signaling, mTOR signaling, and MAPK signaling were identified among the most significantly enriched pathways (FDR < 0.05). Bubble size represents the number of genes associated with each pathway, whereas color intensity indicates the false discovery rate (FDR).

Kyoto Encyclopedia of Genes and Genomes (KEGG) pathway enrichment analysis revealed significant enrichment of several cancer-related signaling pathways (FDR < 0.05). Among the enriched pathways, apoptosis exhibited the highest fold enrichment (8.5-fold; FDR = 0.0001; *n* = 45), followed by the p53 signaling pathway (7.2-fold; FDR = 0.0002; *n* = 38), PI3K–Akt signaling pathway (6.8-fold; FDR = 0.0005; *n* = 52), mTOR signaling pathway (5.9-fold; FDR = 0.0010; *n* = 31), and MAPK signaling pathway (5.4-fold; FDR = 0.0020; *n* = 48) (Figure 14 and Figure 15).

## 3. Discussion

The present study supports our hypothesis that the combination of TQ + DTX exerts enhanced anticancer activity in MDA-MB-231 TNBC cells through coordinated modulation of multiple cellular processes. Compared with either monotherapy, the combination treatment produced greater cytotoxicity, apoptosis induction, intracellular ROS accumulation, fluorescence findings consistent with increased oxidative stress, caspase-9 immunoreactivity, suppression of wound closure, and differential regulation of the apoptosis-related genes *BCL2*, *BAX*, and *CASP9*, together with changes in the expression of the PI3K/AKT pathway-related genes *PTEN*, *PIK3CA*, and *AKT1*. Importantly, NAC pretreatment significantly attenuated ROS accumulation and partially reversed the cytotoxic and pro-apoptotic effects of the combination treatment, further supporting a functional role for oxidative stress in mediating its antitumor activity. Furthermore, bioinformatic analyses identified apoptosis, p53 signaling, PI3K/AKT signaling, mTOR signaling, and MAPK signaling among the pathways potentially associated with the observed gene expression changes. Collectively, these complementary experimental findings provide converging evidence supporting the preclinical potential of the TQ + DTX combination as a therapeutic strategy for TNBC. These observations further reinforce growing evidence that naturally derived bioactive compounds may enhance the efficacy of conventional chemotherapeutic agents while potentially reducing treatment-associated toxicity [14,15,16].

The cytotoxic activity observed following TQ treatment alone is consistent with previous reports demonstrating that TQ induces apoptosis through activation of the intrinsic mitochondrial pathway and disruption of the *BCL2*/*BAX* balance in breast cancer cells [14]. In the present study, the marked downregulation of *BCL2* and concomitant upregulation of *BAX* are consistent with the involvement of mitochondrial apoptotic signaling. Importantly, DTX and TQ appear to target complementary cellular processes. While DTX promotes mitotic catastrophe through microtubule stabilization and prolonged G2/M arrest, TQ enhances mitochondrial dysfunction and oxidative stress, thereby amplifying apoptotic signaling [15,16]. The resulting disruption of mitochondrial membrane integrity has been reported to facilitate cytochrome c release and subsequent activation of downstream caspase cascades.

The Chou-Talalay analysis revealed strong synergism (CI < 0.90) across multiple effect levels, suggesting that simultaneous targeting of distinct molecular pathways contributes to enhanced anticancer efficacy. Similar synergistic interactions have previously been reported for TQ combinations with gemcitabine, methotrexate, and doxorubicin [15,17,18]. Our findings extend these observations to DTX and TNBC models. Furthermore, TQ has been reported to reverse multidrug resistance phenotypes through modulation of drug transport mechanisms and enhancement of intracellular chemotherapeutic accumulation, which may further contribute to the observed synergistic response [19].

A particularly noteworthy finding is the substantial increase in intracellular ROS production following combination treatment. Elevated ROS levels are capable of inducing oxidative DNA damage, mitochondrial depolarization, and activation of apoptosis-related signaling pathways [20,21]. The protective effects observed following NAC pretreatment suggest that oxidative stress contributes to the cytotoxic effects of the TQ + DTX combination. Similar NAC-dependent rescue effects have been documented in other natural compound–chemotherapy combinations [22]. According to Trachootham et al. [23], selective ROS induction may push cancer cells beyond their oxidative stress threshold, thereby triggering apoptosis while sparing normal cells. The approximately 5.0-fold increase in ROS observed in MDA-MB-231 cells is consistent with the involvement of oxidative stress in the apoptotic response induced by the TQ + DTX combination.

The increased caspase-9 immunoreactivity further supports the involvement of the intrinsic apoptotic pathway. Caspase-9 is activated following cytochrome c release and apoptosome formation and serves as a critical initiator of mitochondrial apoptosis [24,25]. The strong caspase-9 immunoreactivity observed in the combination group, together with increased CASP9 expression, is consistent with the involvement of mitochondrial apoptotic signaling. Together with the ROS rescue experiments, these findings support the involvement of a ROS-associated mitochondrial apoptotic mechanism. These findings are consistent with the classical apoptotic model proposed by Li et al. [24], in which caspase-9 activation is described as an upstream event leading to downstream executioner caspase signaling and irreversible commitment to apoptosis. However, the present study evaluated caspase-9 immunoreactivity and *CASP9* gene expression rather than caspase-9 activation directly.

Beyond its cytotoxic activity, the TQ + DTX combination significantly reduced wound closure in MDA-MB-231 cells. Since metastatic dissemination remains the leading cause of cancer-related mortality, inhibition of cellular migration represents an important therapeutic objective [26,27]. The marked reduction in wound closure observed in the combination group indicates impaired wound closure under the experimental conditions used in this study. Previous studies have shown that TQ inhibits invasion and metastasis through modulation of matrix metalloproteinases, focal adhesion kinase signaling, and epithelial–mesenchymal transition pathways [28]. Moreover, *MMP-9* expression is regulated by *PI3K/AKT* signaling [29], suggesting that suppression of this pathway may contribute to the reduced wound closure observed in the present study.

Bioinformatic analyses suggest potential involvement of these pathways. KEGG pathway enrichment identified *PI3K/AKT* signaling as one of the most significantly affected pathways, while protein–protein interaction analyses highlighted *PTEN*, *AKT1*, *TP53*, *CASP3*, and *BCL2* as central hub genes. *PTEN* functions as a critical negative regulator of *PI3K/AKT* signaling and is frequently lost or suppressed in aggressive breast cancers [30,31]. The observed *PTEN* upregulation may therefore contribute to reduced proliferative signaling and increased apoptotic susceptibility. Consistent with this interpretation, clinical datasets indicate that elevated *PTEN* expression is associated with improved prognosis and survival in breast cancer patients [32,33]. Furthermore, previous studies have demonstrated that TQ suppresses *NF-κB* and *STAT3* signaling pathways [34], both of which are major regulators of inflammatory cytokine production [35].

The differential response observed between MDA-MB-231 and HaCaT cells is of particular translational significance. While both cell types exhibited reduced viability and increased apoptosis following combination treatment, the magnitude of these effects was considerably greater in TNBC cells. This selectivity may be explained by the elevated basal ROS levels, altered redox homeostasis, and increased metabolic demands characteristic of malignant cells [26]. TQ has been reported to exhibit antioxidant properties in normal tissues while simultaneously promoting oxidative stress-mediated apoptosis in cancer cells, thereby providing a potential therapeutic window [9]. Supporting this concept, Duman et al. [10] demonstrated that TQ protects non-malignant cells against chemotherapy-associated oxidative damage. Such selective activity is highly desirable for future translational applications.

Several limitations of the present study should be acknowledged. First, the findings are based exclusively on in vitro experiments performed in a single TNBC cell line (MDA-MB-231) and therefore require validation across additional TNBC cell lines representing different molecular subtypes, advanced three-dimensional tumor models, and well-designed in vivo studies. Accordingly, the observed synergistic effects of the TQ + DTX combination should be interpreted with caution until confirmed in these complementary preclinical models. Second, although transcriptional alterations in the apoptosis- and PI3K/AKT pathway-related genes *BCL2*, *BAX*, *CASP9*, *PTEN*, *PIK3CA*, and *AKT1* were demonstrated by qRT-PCR, comprehensive protein-level validation of these molecular changes was not performed. Furthermore, although NAC pretreatment attenuated the biological effects of the TQ + DTX combination, these findings should be interpreted with caution because NAC exhibits biological activities beyond ROS scavenging and may interact with multiple cellular processes. Therefore, the present results support the involvement of ROS in the observed synergistic effects but do not definitively establish ROS as the underlying mechanism. Third, HaCaT cells were included as a non-malignant comparator; however, because they are immortalized human keratinocytes rather than tissue-matched normal mammary epithelial cells, the observed differences should be interpreted as differential cellular sensitivity rather than definitive cancer selectivity. Fourth, the wound-healing assay was performed using treatment concentrations selected to evaluate the overall anticancer activity of the tested agents. Consequently, the observed reduction in wound closure may reflect not only impaired cell migration but also treatment-induced cytotoxicity and reduced cell proliferation. Future studies employing subcytotoxic concentrations together with proliferation-controlled conditions, such as mitomycin C treatment, will be necessary to specifically distinguish the contribution of cell migration. In addition, the bioinformatic analyses were exploratory and should be considered hypothesis-generating until supported by further experimental validation. Pharmacokinetic characteristics, bioavailability, and toxicity profiles of the TQ + DTX combination also remain to be fully characterized. Moreover, the concentrations used in the present in vitro experiments were selected to investigate biological mechanisms under controlled experimental conditions and should not be interpreted as directly equivalent to clinically achievable drug exposures. Future pharmacokinetic and pharmacodynamic studies performed under physiologically relevant conditions will therefore be essential to establish the translational applicability of the TQ + DTX combination. Future studies incorporating proteomic and phosphoproteomic analyses, CRISPR/Cas9-mediated gene silencing, patient-derived models, three-dimensional tumor spheroids, and animal models may provide a more comprehensive understanding of the molecular mechanisms underlying the observed synergistic effects. Ultimately, carefully designed preclinical and clinical studies will be required before translation into clinical practice.

## 4. Materials and Methods

### 4.1. Cell Culture

The human TNBC cell line MDA-MB-231 (ATCC^®^ HTB-26™) and the non-malignant human keratinocyte cell line HaCaT (CLS No. 300493) were cultured in Dulbecco’s Modified Eagle Medium (DMEM; Gibco, Waltham, MA, USA) supplemented with 10% fetal bovine serum (FBS; Gibco, Grand Island, NY, USA), 100 U/mL penicillin, and 100 µg/mL streptomycin (Gibco, Grand Island, NY, USA). Cells were maintained in a humidified incubator at 37 °C under an atmosphere of 5% CO_2_. The culture medium was replaced every 2–3 days, and cells were subcultured upon reaching approximately 80% confluence using 0.25% trypsin–EDTA solution (Gibco, Grand Island, NY, USA). All experiments were performed using exponentially growing cells at approximately 70–80% confluence.

### 4.2. Drug Preparation

TQ (TQ; Sigma-Aldrich, St. Louis, MO, USA; CAS No. 490-91-5; purity ≥ 98%) was dissolved in dimethyl sulfoxide (DMSO; Sigma-Aldrich, St. Louis, MO, USA) to prepare a 100 mM stock solution and stored at −20 °C until use. DTX (DTX; Sigma-Aldrich, St. Louis, MO, USA; CAS No. 114977-28-5) was similarly dissolved in DMSO to obtain a 10 mM stock solution and stored at −20 °C. Immediately before each experiment, the stock solutions were diluted with complete culture medium to the desired working concentrations. The final concentration of DMSO in all treatment and vehicle control groups was maintained at ≤0.1% (*v*/*v*). Preliminary experiments confirmed that this DMSO concentration did not significantly affect cell viability.

### 4.3. MTT Assay

Cell viability was determined using the MTT [3-(4,5-dimethylthiazol-2-yl)-2,5-diphenyltetrazolium bromide; Sigma-Aldrich] assay. MDA-MB-231 and HaCaT cells were seeded into 96-well plates at a density of 5 × 10^3^ cells per well and allowed to adhere for 24 h. Cells were subsequently treated with TQ (5, 10, 25, 50, 100, and 250 µM), DTX (1, 2.5, 5, 10, 25, and 50 nM), or their combinations for 48 h. Combination treatments were prepared using a fixed-ratio combination design based on the IC_50_ values determined in MDA-MB-231 cells. For CI analysis, six fixed-ratio combination dose levels were evaluated: 6.1/1.05, 12.2/2.10, 24.3/4.20, 36.5/6.30, 48.6/8.40, and 60.8/10.5 (TQ µM/DTX nM). For the subsequent mechanistic experiments, the IC_50_ concentrations determined in MDA-MB-231 cells (48.6 µM for TQ and 8.4 nM for DTX) were selected because they produced reproducible biological responses while preserving a sufficient viable cell population for the evaluation of apoptosis, ROS generation, immunocytochemistry, migration, and gene expression. In addition, the higher IC_50_ values observed in HaCaT cells indicated lower sensitivity under the same experimental conditions, supporting the use of these concentrations for comparative biological analyses. Following treatment, 10 µL of MTT solution (5 mg/mL in PBS) was added to each well and incubated for 4 h at 37 °C. The resulting formazan crystals were dissolved in 100 µL of DMSO, and absorbance was measured at 570 nm using 630 nm as the reference wavelength with a microplate reader. Cell viability was expressed as a percentage relative to the vehicle-treated control group. IC_50_ values were calculated from dose–response curves using GraphPad Prism software. Drug interactions were evaluated using the Chou–Talalay method with CompuSyn software (version 1.0; ComboSyn Inc., Paramus, NJ, USA) from six fixed-ratio combination dose levels, where CI values < 0.90 indicated synergistic interactions, values between 0.90 and 1.10 indicated additive interactions, and values > 1.10 indicated antagonistic interactions. All experiments were performed in triplicate and repeated independently at least three times.

### 4.4. Annexin V/PI Flow Cytometry

Apoptosis was quantified by flow cytometry using an Annexin V-FITC/PI Apoptosis Detection Kit (BD Biosciences, San Jose, CA, USA) and analyzed with a BD FACSCanto™ II flow cytometer (BD Biosciences). MDA-MB-231 cells were seeded into 6-well plates at a density of 3 × 10^5^ cells per well and allowed to adhere for 24 h before treatment with TQ (48.6 µM), DTX (8.4 nM), or their combination for 48 h. Following treatment, both floating and adherent cells were collected, washed twice with cold phosphate-buffered saline (PBS), and resuspended in 100 µL of 1× Annexin V binding buffer. Subsequently, 5 µL of Annexin V-FITC and 5 µL of PI were added to each sample, followed by incubation for 15 min at room temperature in the dark. After incubation, binding buffer was added according to the manufacturer’s instructions, and samples were immediately analyzed by flow cytometry. A minimum of 20,000 events was acquired for each sample. Cell populations were classified as viable (Annexin V^−^/PI^−^), early apoptotic (Annexin V^+^/PI^−^), late apoptotic/secondary necrotic (Annexin V^+^/PI^+^), or necrotic (Annexin V^−^/PI^+^). Data were analyzed using the instrument software, and total apoptosis was calculated as the sum of the early and late apoptotic cell populations.

### 4.5. Intracellular ROS Analysis and NAC Rescue Experiments

#### 4.5.1. Flow Cytometric Measurement of Intracellular ROS

Intracellular ROS levels were determined using 2′,7′-dichlorodihydrofluorescein diacetate (DCFH-DA; Sigma-Aldrich, St. Louis, MO, USA). MDA-MB-231 cells were treated with TQ (48.6 µM), DTX (8.4 nM), or their combination for 48 h. Following treatment, cells were incubated with 10 µM DCFH-DA for 30 min at 37 °C in the dark, washed twice with PBS, and immediately analyzed by flow cytometry (BD FACSCanto™ II, BD Biosciences). Cell debris was excluded based on forward- and side-scatter (FSC/SSC) characteristics, and doublets were excluded using FSC-A/FSC-H gating. Mean fluorescence intensity (MFI) of the DCF signal was determined from the gated single-cell population using FlowJo software (version 10.8.1; BD Biosciences, Ashland, OR, USA), and values were normalized to the untreated control group. All samples were acquired using identical instrument settings, and intracellular ROS levels were expressed as a fold change in MFI relative to the untreated control group. Because DCFH-DA is a general oxidation-sensitive probe, the measured fluorescence was interpreted as an indicator of overall intracellular oxidative status rather than a specific ROS species.

For the ROS rescue experiments, cells were pretreated with 5 mM NAC (NAC; Sigma-Aldrich, St. Louis, MO, USA) for 1 h prior to drug treatment. After pretreatment, the NAC-containing medium was removed, the cells were washed with PBS, and fresh culture medium containing TQ, DTX, or their combination was added. Cells were subsequently incubated for 48 h under the same experimental conditions, after which intracellular ROS production, cell viability (MTT assay), and apoptosis (Annexin V/PI flow cytometry) were evaluated.

#### 4.5.2. Fluorescence-Based Visualization of Intracellular ROS

ROS accumulation was visualized using the fluorescent probe 2′,7′-dichlorodihydrofluorescein diacetate (DCFH-DA; Sigma-Aldrich, St. Louis, MO, USA). MDA-MB-231 cells were seeded into 6-well plates and cultured overnight at 37 °C in a humidified atmosphere containing 5% CO_2_. Cells were treated with TQ, DTX, or the TQ + DTX combination for 48 h using the previously determined IC_50_ concentrations. For antioxidant rescue experiments, cells were pretreated with N-acetyl-L-cysteine (NAC, 5 mM) for 1 h before drug treatment. The NAC-containing medium was then removed, the cells were washed with PBS, and fresh medium containing TQ, DTX, or the TQ + DTX combination was added for 48 h.

Following treatment, cells were washed twice with phosphate-buffered saline (PBS) and incubated with 10 μM DCFH-DA for 30 min at 37 °C in the dark. After staining, excess dye was removed by washing with PBS. Representative fluorescence images were acquired using an inverted fluorescence microscope (Olympus, Tokyo, Japan) equipped with a FITC filter set. All fluorescence images were captured using identical exposure time, illumination intensity, gain, and camera settings to ensure direct comparison among treatment groups. Fluorescence intensity was quantified using ImageJ software (version 1.54; National Institutes of Health, Bethesda, MD, USA). The same threshold and analysis parameters were applied to all images, and fluorescence intensity was normalized to the untreated control group and expressed as a fold change. For each image, the same analysis parameters were applied to all experimental groups, and fluorescence intensity was normalized to the untreated control group and expressed as a fold change. Because DCFH-DA is a general oxidation-sensitive probe, the measured fluorescence was interpreted as an indicator of overall intracellular oxidative status rather than a specific ROS species. Representative images from three independent experiments are presented.

### 4.6. Immunocytochemical Staining

MDA-MB-231 cells were seeded onto poly-L-lysine-coated glass slides and allowed to adhere for 24 h before treatment with TQ (48.6 µM), DTX (8.4 nM), or their combination for 48 h. Following treatment, cells were fixed with 4% paraformaldehyde (PFA) for 20 min at room temperature, washed with PBS, and permeabilized with 0.1% Triton X-100 for 10 min. Non-specific binding sites were blocked with 1% bovine serum albumin (BSA) in PBS for 1 h at room temperature. Cells were then incubated overnight at 4 °C with a rabbit anti-caspase-9 primary antibody (ab52298, Abcam, Cambridge, UK). After PBS washing, samples were incubated for 1 h at room temperature with an HRP-conjugated goat anti-rabbit IgG secondary antibody (Invitrogen, Thermo Fisher Scientific, Waltham, MA, USA; 1:500). Immunoreactivity was visualized using 3,3′-diaminobenzidine (DAB), followed by hematoxylin counterstaining. Images were acquired using a light microscope equipped with a 20× objective. DAB staining intensity was quantified using the Color Deconvolution plugin in ImageJ software (version 1.54; National Institutes of Health, Bethesda, MD, USA). Immunoreactivity was evaluated using the H-score method [H-score = Σ (Pi × i)], where Pi represents the percentage of positively stained cells and i represents staining intensity (0 = negative, 1 = weak, 2 = moderate, and 3 = strong), yielding a total score ranging from 0 to 300.

### 4.7. Wound Healing (Scratch) Experiment

Cell migratory capacity was evaluated using a wound healing (scratch) assay. MDA-MB-231 cells were seeded into 6-well plates and cultured until approximately 100% confluence. A linear scratch was generated in the cell monolayer using a sterile 200 µL pipette tip, and detached cells were gently removed by washing twice with PBS. Cells were subsequently treated with TQ (48.6 µM), DTX (8.4 nM), or their combination for 48 h. Representative images of the same wound area were acquired immediately after scratching (0 h) and after 48 h of treatment using a phase-contrast inverted microscope. Wound areas were quantified using ImageJ software (version 1.54; National Institutes of Health, Bethesda, MD, USA), and the percentage of wound closure was calculated as follows: [(initial wound area − remaining wound area)/initial wound area] × 100. Data are presented as the mean ± SD of three independent experiments.

### 4.8. qRT-PCR Analysis

Total RNA was isolated from treated MDA-MB-231 cells using TRIzol™ Reagent (Invitrogen, Thermo Fisher Scientific, Waltham, MA, USA) according to the manufacturer’s instructions. RNA concentration and purity were determined using a NanoDrop™ 2000 spectrophotometer (Thermo Fisher Scientific, Waltham, MA, USA), and samples with an A260/A280 ratio of ≥1.9 were considered suitable for further analysis. First-strand cDNA was synthesized from 1 µg of total RNA using the RevertAid First Strand cDNA Synthesis Kit (Thermo Fisher Scientific, Waltham, MA, USA). Quantitative real-time PCR (qRT-PCR) was performed using SYBR™ Green Master Mix (Applied Biosystems, Waltham, MA, USA) on an Applied Biosystems 7500 Fast Real-Time PCR System. The thermal cycling conditions consisted of an initial denaturation at 95 °C for 10 min, followed by 40 cycles of denaturation at 95 °C for 15 s and annealing/extension at 60 °C for 1 min. A melting curve analysis was performed after amplification to verify the specificity of the PCR products. The expression levels of the apoptosis-related genes *BCL2, BAX,* and *CASP9*, together with the PI3K/AKT pathway-related genes *PTEN, PIK3CA,* and *AKT1*, were analyzed. Primer sequences are listed in Table 1. *GAPDH* and *ACTB* (*β-actin*) were used as endogenous reference genes for normalization. Relative gene expression levels were calculated using the 2^−ΔΔCt^ method and expressed as a fold change relative to the untreated control group. The *BAX/BCL2* expression ratio was calculated for each biological replicate using the corresponding normalized expression values, and the mean ratio was subsequently determined. Each experiment was performed using three independent biological replicates.

### 4.9. Bioinformatics Analysis

PPI network analysis was performed using the STRING database (version 11.5; https://string-db.org; accessed on 15 June 2026) with a minimum required interaction score of 0.700 (high confidence). The resulting interaction network was imported into Cytoscape (version 3.9.1) for network visualization and topological analysis. Hub genes were identified and ranked according to their betweenness centrality values. KEGG pathway enrichment analysis was subsequently performed using the ClueGO plugin (version 2.5.8) in Cytoscape together with the DAVID functional annotation database. Pathways with an FDR-adjusted *p* value < 0.05 were considered significantly enriched.

### 4.10. Statistical Analysis

All quantitative data are presented as the mean ± standard deviation (SD) of three independent biological experiments (*n* = 3). Individual biological replicate values are displayed in the corresponding graphs. Statistical analyses were performed using GraphPad Prism version 9.0 (GraphPad Software, San Diego, CA, USA). Comparisons among multiple groups were conducted using one-way analysis of variance (ANOVA) followed by Tukey’s multiple comparisons post hoc test. A *p* value < 0.05 was considered statistically significant.

Cell viability data obtained from the MTT assay were used to generate dose–response curves for the calculation of half-maximal inhibitory concentration (IC_50_) values by nonlinear regression analysis in GraphPad Prism. Drug interactions were evaluated according to the Chou–Talalay method using CompuSyn software (version 1.0; ComboSyn Inc., Paramus, NJ, USA). CI values < 0.90 were considered synergistic, values between 0.90 and 1.10 were considered additive, and values > 1.10 were considered antagonistic. The selectivity index (SI) was calculated as the ratio of the IC_50_ value obtained in HaCaT cells to that obtained in MDA-MB-231 cells.

Flow cytometric analyses, including apoptosis and intracellular ROS measurements; quantitative fluorescence intensity measurements obtained from DCFH-DA fluorescence microscopy; wound closure percentages; immunocytochemical H-score values; and relative gene expression levels determined by qRT-PCR, were statistically analyzed using one-way ANOVA followed by Tukey’s multiple comparisons post hoc test. Relative gene expression levels were calculated using the 2^−ΔΔCt^ method and expressed as a fold change relative to the untreated control group. Quantitative fluorescence intensity, flow cytometry, wound healing, immunocytochemistry, and qRT-PCR data are presented as the mean ± SD of three independent biological experiments. For BAX/BCL2 ratio analysis, the normalized relative expression values (2^−ΔΔCt^) obtained for *BAX* and *BCL2* from each independent biological replicate were used. The BAX/BCL2 ratio was calculated individually for each biological replicate by dividing the normalized *BAX* value by the corresponding normalized *BCL2* value. The resulting replicate-level ratios were then used for statistical analysis and are presented as mean ± SD.

## 5. Conclusions

The present study demonstrates that the TQ + DTX combination exerts enhanced anticancer activity in MDA-MB-231 triple-negative breast cancer cells compared with either agent alone. Combination treatment was associated with increased cytotoxicity, apoptosis induction, intracellular ROS accumulation consistently demonstrated by both flow cytometric and fluorescence-based analyses, increased caspase-9 immunoreactivity, reduced wound closure, and altered mRNA expression of the apoptosis-related genes *BCL2, BAX,* and *CASP9*, together with altered expression of the PI3K/AKT pathway-related genes *PTEN, PIK3CA,* and *AKT1*. Furthermore, NAC pretreatment significantly attenuated ROS accumulation and partially reversed the cytotoxic and pro-apoptotic effects of the combination treatment, suggesting that oxidative stress may contribute to, but does not solely explain, the antitumor activity of the TQ + DTX combination. In addition, bioinformatic analyses identified apoptosis, p53, PI3K/AKT, mTOR, and MAPK signaling as pathways potentially associated with the observed molecular responses. Although these findings are limited to in vitro models, the greater sensitivity observed in MDA-MB-231 cells compared with HaCaT cells provides preliminary evidence of differential cellular responses. Collectively, the present experimental and bioinformatic findings support further preclinical investigation of the TQ + DTX combination as a promising therapeutic strategy for triple-negative breast cancer. However, its translational potential should be interpreted with caution and requires validation in additional TNBC models, advanced three-dimensional tumor systems, and well-designed in vivo studies before clinical application can be considered.

## Figures and Tables

**Table 1:** qRT-PCR Primer Sequences.

Genes	Forward Primer (5′→3′)	Reverse Primer (5′→3′)
*BCL-2*	ATGCCTTTGTGGAACTATATGGC	GGTATGCACCCAGAGTGATGC
*BAX*	CCCGAGAGGTCTTTTTCCGAG	CCAGCCCATGATGGTTCTGAT
*CASP9*	GAAGCGAATCAATGGACTCGG	CTTGCACTCCTGCATCAGCTT
*PTEN*	TGGATTCGACTTAGACTTGACCT	GCGGTGTCATAATGTCTTTCAG
*PIK3CA*	CTCAACTGAGTTCCTTGTTGGC	AACTTCATGGACGATGCACTG
*AKT1*	TGGACTACCTGCACTCGGAGAA	GTGCCGCAAAAGGTCTTCATGG
*GAPDH*	GTCTCCTCTGACTTCAACAGCG	ACCACCCTGTTGCTGTAGCCAA

## Data Availability

The original contributions presented in this study are included in the article. Further inquiries can be directed to the corresponding authors.

## Abbreviations

The following abbreviations are used in this manuscript:

ANOVA	Analysis of Variance
BSA	Bovine Serum Albumin
cDNA	Complementary DNA
CI	Combination Index
DAB	3,3′-Diaminobenzidine
DCFH-DA	2′,7′-Dichlorodihydrofluorescein Diacetate
DMEM	Dulbecco’s Modified Eagle Medium
DMSO	Dimethyl Sulfoxide
DTX	Docetaxel
Fa	Fraction Affected
FBS	Fetal Bovine Serum
FDR	False Discovery Rate
HRP	Horseradish Peroxidase
IC_50_	Half-Maximal Inhibitory Concentration
KEGG	Kyoto Encyclopedia of Genes and Genomes
MTT	3-(4,5-Dimethylthiazol-2-yl)-2,5-Diphenyltetrazolium Bromide
NAC	N-Acetyl-L-Cysteine
PBS	Phosphate-Buffered Saline
PFA	Paraformaldehyde
PI	Propidium Iodide
PPI	Protein–Protein Interaction
qRT-PCR	Quantitative Real-Time Polymerase Chain Reaction
RNA	Ribonucleic Acid
ROS	Reactive Oxygen Species
SD	Standard Deviation
SI	Selectivity Index
TNBC	Triple-Negative Breast Cancer
TQ	Thymoquinone
